# Identification of an SCPL Gene Controlling Anthocyanin Acylation in Carrot (*Daucus carota* L.) Root

**DOI:** 10.3389/fpls.2019.01770

**Published:** 2020-01-31

**Authors:** Julien Curaba, Hamed Bostan, Pablo F. Cavagnaro, Douglas Senalik, Molla Fentie Mengist, Yunyang Zhao, Philipp W. Simon, Massimo Iorizzo

**Affiliations:** ^1^ Plants for Human Health Institute, North Carolina State University, Kannapolis, NC, United States; ^2^ National Scientific and Technical Research Council (CONICET), Instituto Nacional de Tecnología Agropecuaria (INTA) E.E.A., La Consulta, Mendoza, Argentina; ^3^ Facultad de Ciencias Agrarias, Universidad Nacional de Cuyo, Mendoza, Argentina; ^4^ Department of Horticulture, University of Wisconsin–Madison, Madison, WI, United States; ^5^ Vegetable Crops Research Unit, US Department of Agriculture–Agricultural Research Service, Madison, WI, United States; ^6^ Department of Horticultural Science, North Carolina State University, Raleigh, NC, United States

**Keywords:** *Daucus carota* L., anthocyanins, acyltransferase, fine mapping, transcriptome, candidate genes

## Abstract

Anthocyanins are natural health promoting pigments that can be produced in large quantities in some purple carrot cultivars. Decoration patterns of anthocyanins, such as acylation, can greatly influence their stability and biological properties and use in the food industry as nutraceuticals and natural colorants. Despite recent advances made toward understanding the genetic control of anthocyanin accumulation in purple carrot, the genetic mechanism controlling acylation of anthocyanin in carrot root have not been studied yet. In the present study, we performed fine mapping combined with gene expression analyses (RNA-Seq and RT-qPCR) to identify the genetic factor conditioning the accumulation of non-acylated (Cy3XGG) *versus* acylated (Cy3XFGG and Cy3XSGG) cyanidin derivatives, in three carrot populations. Segregation and mapping analysis pointed to a single gene with dominant effect controlling anthocyanin acylation in the root, located in a 576kb region containing 29 predicted genes. Orthologous and phylogenetic analyses enabled the identification of a cluster of three SCPL-acyltransferases coding genes within this region. Comparative transcriptome analysis indicated that only one of these three genes, *DcSCPL1*, was always expressed in association with anthocyanin pigmentation in the root and was co-expressed with *DcMYB7*, a gene known to activate anthocyanin biosynthetic genes in carrot. *DcSCPL1* sequence analysis, in root tissue containing a low level of acylated anthocyanins, demonstrated the presence of an insertion causing an abnormal splicing of the 3^rd^ exon during mRNA editing, likely resulting in the production of a non-functional acyltransferase and explaining the reduced acylation phenotype. This study provides strong linkage-mapping and functional evidences for the candidacy of *DcSCPL1* as a primary regulator of anthocyanin acylation in carrot storage root.

## Introduction

Anthocyanins are water-soluble pigments responsible for the vibrant red-to-blue color commonly found in many organs of higher plant species, including flowers and fruits, which contribute to attract animals and insects for seed dispersal and pollination (Koes et al., 2005). In plants, anthocyanins are proposed to play a protective role when accumulating in response to oxidative stresses following UV exposure, cold, drought, phosphate deficiency, and leaf senescence (Dixon and Paiva, 1995; Gould et al., 2010; Zhang et al., 2010; Zhang et al., 2012; Kovinich et al., 2014; Lotkowska et al., 2015; Xu et al., 2017a). Anthocyanins are used as natural colorants in the food industry, and the potential health benefits associated with their antioxidant and anti-inflammatory properties has brought them additional attention (Giusti and Wrolstad, 2003; Akhtar et al., 2017). Indeed, anthocyanin-rich diets have been associated with lower incidence of chronic diseases, including cardiovascular disease, diabetes, arthritis, neurological disorders, and some types of cancers (Cho et al., 2004; Khoo et al., 2017; Ullah et al., 2019).

Anthocyanins are produced through the flavonoid pathway and stored in the vacuole (Koes et al., 2005). Their core structure is composed of an aglycone backbone (C6-C3-C6) known as anthocyanidins, with cyanidin, delphinidin, pelargonidin, peonidin, petunidin, and malvidin being the anthocyanidins most commonly found in nature (Ramos et al., 2014). Various patterns of glycosylation and acylation of the aglycone backbone can affect the chemical properties of anthocyanins, including cellular transport, stability, and bioavailability (Prior and Wu, 2006). Clinical human feeding studies using raw and cooked purple carrots revealed that non-acylated anthocyanins are more bioavailable than their acylated counterpart (Kurilich et al., 2005; Charron et al., 2009; Novotny et al., 2012). Acylation of anthocyanins, in contrast, promotes their color stability, and consequently their application as natural food colorants (Malien-Aubert et al., 2001; Giusti and Wrolstad, 2003; Kammerer et al., 2004). Thus, the relative content of acylated and non-acylated anthocyanin forms directs their utilization in the food industry as nutraceuticals and natural colorants.

**Figure 1:**
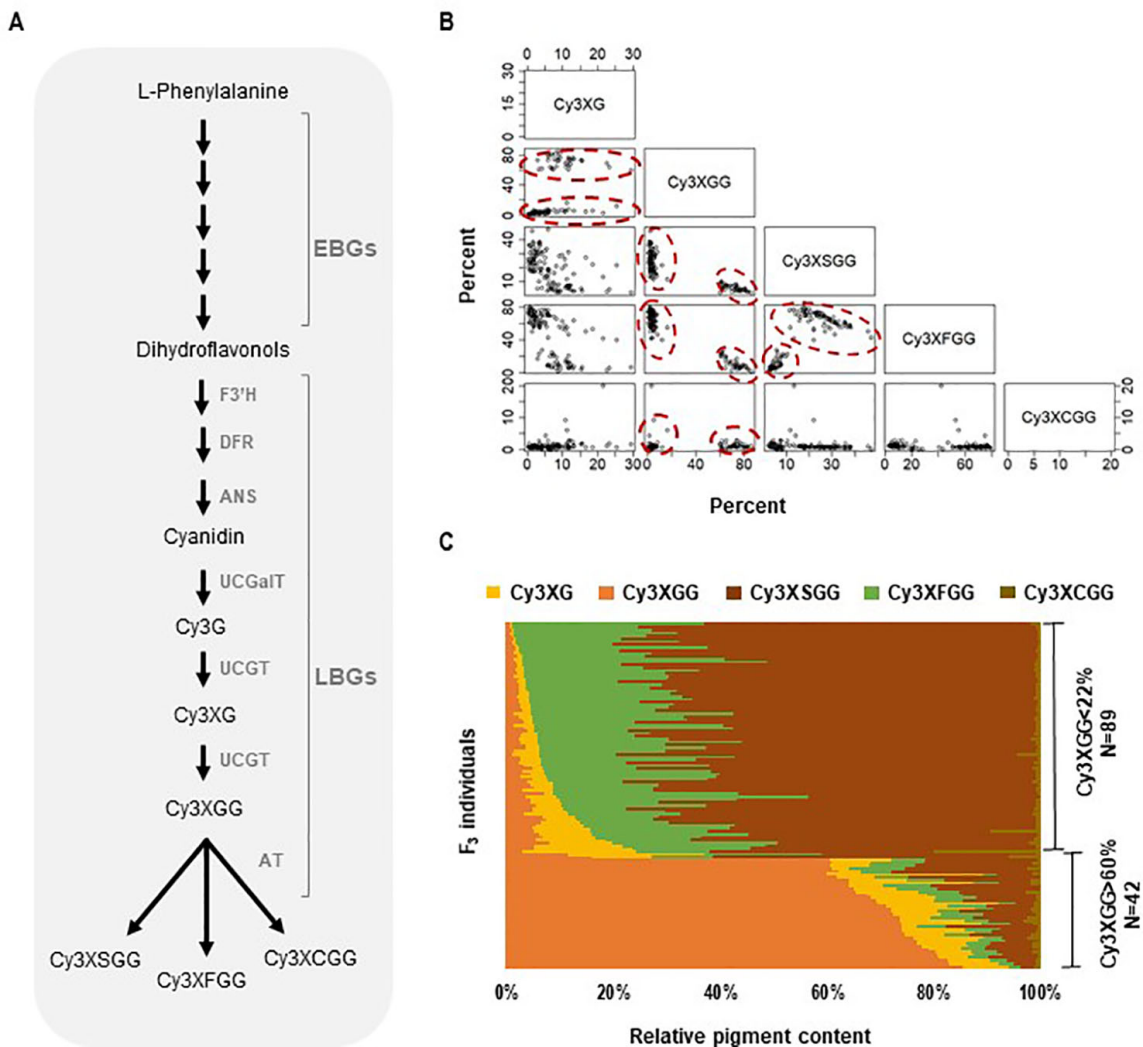
Anthocyanin biosynthesis and composition of cyanidin derivatives in the 5394 population. **(A)** Anthocyanin biochemical pathway in carrot involving early and late biosynthesis genes (EBGs and LBGs, respectfully). *F3′H, flavonoid 3′-hydroxylase*; *DFR, dihydroflavonol 4-reductase*; *ANS, anthocyanidin synthase*; *UCGalT*; *UCGT, UDP-glucose: cyanidin-3-O-glucosyltransferase; AT, acyltransferase*. **(B, C)** Relative content and correlation analysis among cyanidin derivatives in the 5394 population. HPLC analysis was performed on plants with root purple pigmentation (*N* = 131) to quantify the following cyanidin derivatives: Cy3XG, Cy3XGG, Cy3XSGG, Cy3XFGG, and Cy3XCGG. **(B)** Scatter plot for pairwise correlations among the five cyanidin derivatives. Circles indicate individuals with Cy3XGG < 22% (HAA) and Cy3XGG > 60% (LAA). **(C)** Relative content (%) of five cyanidin derivatives in 131 purple-rooted individuals of population 5394.

Purple carrots can accumulate large quantities of anthocyanins in their roots (Mazza and Miniati, 1993; Montilla et al., 2011) and in the recent years, black carrots have received much attention as a natural source of anthocyanins for coloring fruit juices, soft drinks, jellies, and confectioneries (Akhtar et al., 2017). Cyanidin is the major type of anthocyanidin accumulating in the storage root of purple carrot (Kammerer et al., 2004; Montilla et al., 2011; Khoo et al., 2017), although pelargonidin and peonidin glycosides have also been reported in trace quantities in some genetic stocks (Kammerer et al., 2003). The formation of cyanidin-3-galactoside (Cy3G), is catalyzed by a UDP-galactose:cyanidin galactosyltransferase (*DcUCGalT*) (Xu et al., 2016), which is further glycosylated into cyanidin-3-(2”-xylose-galactoside) (Cy3XG) and cyanidin-3-(2”-xylose-6-glucose-galactoside) (Cy3XGG), the two main forms of non-acylated anthocyanins (**Figure 1A**). Cy3XGG is then acylated through transesterification, using sinapoyl, feruloyl, or p-coumaroyl esters as donors, to form cyanidin-3-(2”-xylose-6”-sinapoyl-glucose-galactoside) (Cy3XSGG), cyanidin-3-(2”-xylose-6”-feruloyl-glucose-galactoside) (Cy3XFGG), or cyanidin-3-(2”-xylose-6”-(4-coumuroyl)glucose-galactoside) (Cy3XCGG), respectively (Alasalvar et al., 2001; Algarra et al., 2014). In all the purple carrot cultivars studied, Cy3XSGG and Cy3XFGG are always the two major forms of acylated anthocyanins present in the storage root (Montilla et al., 2011; Cavagnaro et al., 2014). However, depending on the carrot genetic background, the percentage of acylated anthocyanins relative to the total anthocyanins can vary from 25 to 85% (Mazza and Miniati, 1993; Kammerer et al., 2004; Cavagnaro et al., 2014). Black carrots accumulate a high percentage of mono-acylated forms of anthocyanins (Giusti and Wrolstad, 2003; Kammerer et al., 2004) and unlike radishes and red cabbage, their use as food colorants does not require the removal of the typical sulfur aroma found in the latter sources of acylated anthocyanins (Giusti and Wrolstad, 2003).

The genetic regulation of the anthocyanin biosynthesis pathway, which requires the coordination of early and late biosynthetic genes (EBGs and LBGs, respectively) in the pathway, is highly conserved across the plant kingdom (Campanella et al., 2014). Upregulation of anthocyanin-related LBGs expression is generally observed between purple and non-purple tissues, as opposed to EBG’s expression which does not consistently correlates with the production of anthocyanins, indicating that LBGs are probably more specific of the anthocyanin biosynthesis pathway than EBGs (Liu et al., 2018). The transcriptional regulation of anthocyanin structural genes, especially LBGs, is controlled by the MYB-bHLH-WD40 protein complex, in which the specificity of MYB transcription factors is critical in coordinating the activation or repression of the anthocyanin biosynthesis pathway (Lin-Wang et al., 2010; Shi and Xie, 2014; Zhang et al., 2014; Liu et al., 2018; Naing and Kim, 2018).

Acyltransferases are among the last LBGs involved in the anthocyanin biosynthesis pathway. These enzymes have a high substrate specificity for both anthocyanin acceptors and acyl group donors (He and Giusti, 2010). Acyltransferases known to modify phenolic compounds were found in two distinct protein families, BAHD and SCPL (Serine Carboxypeptidase-Like) (Mugford and Milkowski, 2012; Bontpart et al., 2015). SCPL-acyltransferases (SCPL-ATs) share homology with the Peptidase_S10 family domain (Mugford and Milkowski, 2012). SCPL-ATs, unlike classical hydrolase of the SCP family, catalyze a transesterification reaction using 1-O-β-glucose esters as their activated donor, whereas BAHD uses acyl-CoA thioesters (Bontpart et al., 2015). In *Arabidopsis*, five SCPL-ATs using sinapoylglucose as the activated donor, have been characterized: AtSCPL8 (SMT, sinapoyl:malate transferase), AtSCPL19 (SCT, sinapoyl:choline transferase), AtSCPL9 and AtSCPL13 (SSTs, sinapoyl:sinapoyl transferases), and AtSCPL10 (SAT, sinapoyl:anthocyanin transferase), where AtSCPL10 is the only characterized SCPL demonstrated to use an anthocyanin as its acyl acceptor substrate (Lehfeldt et al., 2000; Shirley et al., 2001; Shirley and Chapple, 2003; Fraser et al., 2007; Fraser and Chapple, 2011). Crude enzymes extracted from purple carrot taproots exhibited UDP-glucose: sinapic acid glucosyltransferase activity, which catalyzes the formation of sinapoylglucose, and an acyltransferase activity, which uses 1-O-β-glucose esters as acyl donors to catalyze the formation of acylated anthocyanins (GlaBgen and Seitz, 1992; Chen et al., 2016).

In purple carrot, progress has been made toward understanding the genetic control of anthocyanin accumulation. Four major loci (*P1, P3, RTPE-Q1*, and *RTPE-Q2*) controlling anthocyanin accumulation in carrot roots have been mapped (Yildiz et al., 2013; Cavagnaro et al., 2014; Iorizzo et al., 2019). Using a fine mapping approach, we recently identified a cluster of six anthocyanin related R2R3-MYB transcription factors coding genes in the region of *RTPE-Q1* (Iorizzo et al., 2019). Two of these MYBs, *DcMYB6* and *DcMYB7*, were functionally characterized and shown to promote anthocyanin biosynthesis in *Arabidopsis* and carrot, respectively (Xu et al., 2017b; Xu et al., 2019).

To date, however, no gene controlling the acylation of anthocyanins has been characterized in carrot. Using a carrot F2 population segregating for purple pigmentation, named 70349, we previously mapped the *Root Anthocyanin Acylation 1* (*Raa1*) locus controlling high *versus* low acylated anthocyanin content in the storage root (Cavagnaro et al., 2014). In the present study, we used 70349 and three additional populations to fine map the *Raa1* locus to a 576 kb region. Phylogenetic and transcriptome analysis pointed to one candidate gene, named *DcSCPL1*, coding for a potential acyltransferase. *DcSCPL1* expression is up-regulated in purple roots and co-expressed with *DcMYB7*. Sequence analysis of *DcSCPL1* in both high and low acylated backgrounds, revealed the presence of two distinct alleles, *DcSCPL1.1* and *DcSCPL1.2*, and strongly suggests that *DcSCPL1* is a major player in the accumulation of Cy3XSGG and Cy3XFGG in the carrot storage root.

## Materials and Methods

### Plant Materials

Inheritance and fine mapping of the *Raa1* locus was studied in four segregating populations (two F_2_s and two F_3_s) for a total of 956 phenotyped plants. Population 70349 is an F_2_ family (*N* = 497) derived from an initial cross between P4201 and B6320. P4201 is an inbred line with purple outer phloem and yellow xylem storage roots, and purple petioles that was derived from a cross between inbred P9547, with purple xylem and phloem root color derived from Central Anatolia (Turkey), and B2566, an inbred with orange root color from diverse European sources. B6320 is an inbred with orange roots and green petioles derived from the European open-pollinated cultivars Nantes and Camberly. Populations 5392 (*N* = 150) and 5394 (*N* = 171) are F_3_ families segregating for purple root color derived from self-pollination of two 70349 F_2_ plants with purple roots.

Population 7095 (*N* = 138) is a test-cross F_2_ population derived from an initial cross made between two plants with purple roots, BP5394-1 and BP5748-7. These two plants were selected based on their allelic status at the acylation locus (*Raa1*). BP5394-1 was a purple-rooted plant selected from the 5394 F_3_ family described above, and homozygous recessive at the *Raa1* locus. BP5748-7 was a purple-rooted carrot derived from PI652188 from China (also known as Ping Ding), and homozygous dominant at the *Raa1* locus. These plants were selected based on HPLC profile and *DcSCPL1* sequence analysis. Growing conditions and phenotyping for these populations were previously described by Cavagnaro et al. (2014) and Iorizzo et al. (2019). Briefly, seeds from the four populations were grown at the University of California Desert Research and Extension Center (UC-DREC; Holtville, California) in the growing season of 2013–2014, using conventional agricultural practices for carrots.

In addition to these populations, roots grown at UC-DREC from populations 7280, 8519, and 95710 were used for transcriptome analysis. Population 7280 is an F_2_ family derived from a Turkish purple carrot not closely related to the parents of population 70349. Population Y8519 is an F_3_ family from the same genetic background as population 70349 but it does not accumulate anthocyanins and segregates for yellow and orange root colors. Population 95710 is an F_2_ family derived from a Syrian purple carrot intercrossed with an orange carrot of European origin.

### Phenotypic, HPLC, and Segregation Analysis

Phenotypic data for root pigmentation in the 70349, 5392, and 5394 populations was previously described by Cavagnaro et al. (2014) and Iorizzo et al. (2019) to study the genetic mechanism controlling total anthocyanin accumulation in carrot roots. The roots of all the 7095 population plants were purple. High-performance liquid chromatography (HPLC) analysis for the 70349 population was described in these studies. For the populations 7095, 5392, and 5394, root tissues were lyophilized and anthocyanins were extracted with acidified methanol, followed by HPLC analysis as described by Kurilich et al. (2005). The five major carrot anthocyanin pigments (all cyanidin derivatives) were identified and quantified in all four populations (**Supplementary Table S1**). For QTL mapping the HPLC data were expressed as percentage concentration of a given pigment relative to the total anthocyanin content, which was derived from the sum of the content of the five individual anthocyanin compounds, as described previously (Cavagnaro et al., 2014).

To analyze the inheritance of the *Raa1* locus and map it, individuals with more than 60% of Cy3XGG were scored as “low acylated anthocyanin” (LAA) and those with less than 22% of Cy3XGG were scored as “high acylated anthocyanin” (HAA).

### Fine Mapping of QTL Conditioning the *Raa1* Locus in the 70349, 5392, and 5394 Populations

HPLC data and genotypic scores for 38 SNP markers located on chromosome 3 and covering the *Raa1* map region were obtained for 418 individuals from population 70349 [including 231 that were genotyped in the present study and 187 previously genotyped by Cavagnaro et al. (2014)], and were used for linkage map construction and QTL analysis. In addition, a new SNP marker located in the coding region of *DcUCGalT1*, a gene responsible for anthocyanin galactosylation in purple carrot (Xu et al., 2016), and located in the vicinity of *Raa1*, was developed and used to genotype individuals from the 70349 population. A subset of 11 SNP markers were used to map the *Raa1* locus in the 5392 and 5394 populations.

Total genomic DNA of individual plants was isolated from lyophilized leaves following the protocol described by Murray and Thompson (1980) and quantified using Quant-iT™ PicoGreen^®^ (Invitrogen, Paisley, UK). Genotyping was performed using KASPar Chemistry (https://www.lgcgroup.com/products/kasp-genotyping-chemistry/#.Wx5-8novxjU) as previously described by Iorizzo et al. (2011). Primer sequences used to generate SNP scores and map the locus are provided in **Supplementary Tables S2** and **S3**. SNP scores were converted into genotype codes using the A/H/B system for co-dominant markers segregating in an F_2_ population. JoinMap 4.0 software (Ooijen, 2006) was used for mapping, as previously described by Cavagnaro et al. (2014). QTL analysis was performed using R/qtl with the multiple imputations method (Broman and Sen, 2009). QTL detection included preliminary QTL identification using “scanone” followed by QTL modeling. The largest LOD peak from the analysis was added to the QTL model and if the QTL model was significant, it was retained. This process was then repeated using “addqtl,” instead of “scanone,” followed by QTL modeling and testing for interactions until adding additional QTL to the model was no longer significant. The support intervals were calculated using a 1.5 LOD drop. The *Raa1* locus was mapped as a simply inherited dominant trait (Cavagnaro et al., 2014), scoring the individuals with more than 60% of Cy3XGG (coded as “A”) and those with less than 22% of Cy3XGG (coded as “C”). Mapping was performed in 70349, 5392, and 5394 populations.

### Identification and Analysis of Candidate Acyltransferase Coding Genes in Carrot

Sequences corresponding to the SNP markers flanking, and located within, the *Raa1* support interval in population 70349, and the SNP markers flanking the *Raa1* locus in the populations 5392 and 5394 linkage maps, were aligned to the carrot genome assembly (Iorizzo et al., 2016) to determine the physical distance, in terms of number of nucleotides, amongst them. After this, SNP genotypic scores for these markers in the individuals of each population were used to establish haplotype blocks and to identify recessive (*aa*) to dominant (*A_*) recombinants (crossover point) spanning this region.

A collection of 32,113 genes predicted in the carrot genome assembly DCARv2 (Iorizzo et al., 2016) and 6,626 RefSeq (NCBI Reference Sequences) gene models, not overlapping with any gene predicted in DCARv2, were used to perform an orthologous and phylogenetic analysis against 122 characterized and putative members of the SCP/SCPL and BAHD gene families from numerous plant species (**Supplementary Table S4**). Orthologous analysis was performed using the OrthoMCL pipeline (Li et al., 2003) with an inflation value (-I) of 1.5. Genes clustered in orthologous groups with at least one SCP, SCPL, or BAHD gene were extracted and used for the downstream analysis. Phylogenetic analysis was conducted using MEGA version 7.0.26 (Kumar et al., 2016) and the protein sequences from the different orthologous genes. Multiple sequence alignment of the resulting gene set was performed using Muscle 3.6 (Edgar, 2004), with default parameters. Evolutionary distances were computed using the Poisson correction method and a Neighbor-joining (NJ) method, with 1,000 bootstrap replicates, was used to construct the phylogenetic tree. All positions containing gaps and missing data were eliminated from the data set (complete deletion option). Lists of plant genomes and genes used for these analyses are presented in **Supplementary Tables S4** and **S5**.

### Transcriptome Analysis

RNA-Seq data was extracted from two recent studies published by our group (Bannoud et al., 2019; Iorizzo et al., 2019). Seven carrot root tissue types representing different morphological and biochemical phenotypes were used for comparative transcriptome analysis. Four purple-rooted carrot lines were evaluated: (1) a purple root with high acylated anthocyanins from the population 5394 (5394-PR-HAA); (2) a purple root with low acylated anthocyanins from the population 5394 (5394-PR-LAA); (3) a purple root from the population 95710 (95710-PR); (4) a purple root with high acylated anthocyanins from the population 7280-F2 (7280-PR-HAA). Three non-purple-rooted carrot lines were evaluated: (5) an orange root from the population 5394 (5394-OR); (6) an orange root from the population Y8519-F3 (8519-OR); (7) a yellow root from the population Y8519-F3 (8519-YR). Three biological replicates (i.e., roots tissue from three plants) were sampled for each line/phenotype. Detailed statistics on the RNA-Seq tissue/phenotype samples, including read processing and mapping, and comparisons performed are presented in **Supplementary Tables S6** and **S7**.

Reads were filtered with Trimmomatic (Bolger et al., 2014), considering TruSeq adapters 2:30:10 LEADING:3 TRAILING:3 SLIDINGWINDOW:4:15 MINLEN:36. In this process, adapter sequences were removed from the reads and low-quality bases were trimmed from the 3’ end of the reads. The quality check of the remaining sequences was performed using FastQC (Andrews, 2010). High-quality short reads from each replicate were independently mapped against the carrot genome sequence (GenBank accession LNRQ01000000.1) using STAR version 020201 (Dobin et al., 2013), considering the following parameters: –alignEndsType = EndToEnd; –outFilterMismatchNmax = 2; –outFilterMultimapNmax = 20. Reads for each gene available from the V1.0 gene annotation of the carrot genome (Iorizzo et al., 2016) as well as the RefSeq novel gene models, not predicted in V1.0 (6,626 genes), were quantified with the featureCounts standalone package (Liao et al., 2014), using only reads that mapped uniquely to the genome.

Total read abundance for each gene and each biological replicates was calculated by combining the read counts for a given locus from each technical replicate. Estimation of variance-mean dependence in count data and the test for the differentially expressed genes (DEGs) were based on the General Linear Model (GLM) implemented in the EdgeR package (Robinson et al., 2010), considering False Discovery Rate (FDR) ≤ 0.05. Pearson correlation values between samples and technical replicates were calculated and samples that correlated with non-corresponding biological or technical replicates were discarded.

### Real-Time Quantitative Reverse Transcriptase PCR

Total RNA was extracted from twelve-week old root tissue using Trizol reagent (Invitrogen, Carlsbad, CA, United States) and treated with RNase-free DNase I (New England BioLabs, Ipswich, MA, United States). First-strand cDNA synthesis was performed on 1 μg of total RNA using the SuperScript™ III First-Strand Synthesis System (Invitrogen, Carlsbad, CA, United States). Quantitative PCR (qPCR) reactions were carried out in 10 μl final volume containing 10 ng of cDNA, 5 pmol of each primers, and 5 μl of PowerUp™ SYBR™ Green Master Mix (Applied Biosystems, Foster City, CA, United States). The reactions were run in triplicates in an ABI 7500 Sequence Detection System using the following program: 95°C for 2 min, followed by 40 cycles at 95°C for 15 sec, 55°C for 15 sec, and 72°C for 1 min. Melting curves were analyzed for each primer set. Primers information is provided in **Supplementary Table S8**. The *Actin* gene was used as an internal control to calculate the relative expression levels of each gene of interest by the 2^-ΔCT^ method and multiplied by 1,000 (formula 1,000 * 2^-ΔCT^) to enhance readability (Livak and Schmittgen, 2001; Tian et al., 2015). Statistical comparisons were done through one-way analysis of variance (ANOVA) using the software Statistical Product and Service Solutions (SPSS) v 23 (IBM, NY) followed by Tukey’s HSD test to determine the significant difference results.

### Sequencing and Genotyping of *DcSCPL1* Alleles

Total genomic DNA and RNA of six population 5394 plants with contrasting biochemical phenotypes and haplotypes (homozygous LAA and homozygous HAA), was isolated and evaluated as described above. Primers flanking the genomic location of the *DcSCPL1* gene were designed using Primer3 (Koressaar and Remm, 2007; Untergasser et al., 2012) and are listed in **Supplementary Table S8**. The primers 12-5F/12-1R were used for amplifying and sequencing *DcSCPL1* cDNA using Sanger sequencing as described by Iorizzo et al. (2011). The primers 12-2F/12-2R were used for the amplification and sequencing of the genomic region harboring exon-3, as well as for the genotyping of *DcSCPL1* alleles using Taq DNA Polymerase with ThermoPol^®^ Buffer (New England BioLabs, Ipswich, MA, United States) and the following PCR program: 94°C for 2 min, followed by 30 cycles at 94°C for 15 sec, 50°C for 15 sec, and 68°C for 2 min 30 sec, and a final elongation at 68°C for 5 min.

## Results

### Pigment Analysis and Inheritance

Five cyanidin derivatives accumulated in the purple roots of all four segregating populations; two non-acylated (Cy3XG, Cy3XGG) and three acylated (Cy3XFGG, Cy3XSGG, Cy3XCGG) pigments (**Supplementary Table S1**). Across all four populations, two clear phenotypes were identified, one group with individuals having less than 22% of Cy3XGG and another group having more than 60% of Cy3XGG (**Figures 1B, C** **and** **Supplementary Figures S1** and **S2**) consistent with the pattern observed by Cavagnaro et al. (2014). In all populations, all individuals with low percentage of Cy3XGG (< 22%) had high percentage of the combined acylated anthocyanins Cy3XSGG and Cy3XFGG (from 50 to 99%); while all individuals with high percentage of Cy3XGG (> 60%) had low percentage of these two acylated anthocyanins (from 4 to 36%) (**Figures 1B, C** and **Supplementary Figures S1** and **S2**). Among individuals with high content of acylated anthocyanins (i.e., those with <22% of Cy3XGG), the feruloyl-containing pigment (Cy3XFGG) was the most abundant anthocyanin in most plants, with a relative concentration range of 25–87%, followed by Cy3XSGG (9–60%), and Cy3XCGG (0–5%). Correlation analysis based on the percentage of each anthocyanin across all individuals and populations indicated that non-acylated anthocyanins were strongly and inversely correlated with acylated anthocyanins (r = −0.59 to −1.00, P < 0.05; **Supplementary Table S9**). Among samples with high content of acylated anthocyanins, the percentage of Cy3XFGG was inversely correlated with the percentage of Cy3XSGG (r = −0.47 to 0.73, P < 0.05; **Supplementary Table S9**) as well as with the percentage of both Cy3XG and Cy3XGG (r = −0.34 to −0.77, P < 0.05; **Supplementary Table S9**). The percentage of Cy3XCGG was negatively correlated with Cy3XSGG (r = −0.31 to −0.56; P < 0.05; **Supplementary Table S9**) but did not correlate with any of the other anthocyanins.

Two clear groups of 25, 42, 47, and 36 individuals with low acylated anthocyanins “LAA” (> 60% of Cy3XGG) and 68, 89, 192, 102 individuals with high acylated anthocyanins “HAA” (< 22% of Cy3XGG) were identified in 5392, 5394, 70349, and 7095 populations, respectively (**Supplementary Figure S1**). These results suggest that acylation of Cy3XGG, to produce Cy3XSGG and Cy3XFGG, results in the shift from LAA to HAA content and a relatively simple genetic basis underlies the acylation. To test this hypothesis, a chi-square analysis was performed by scoring the samples with LAA and HAA phenotypes. For all populations, the HAA : LAA ratio fit a single gene model (**Table 1**). The LAA and HAA classification, was used to score the *Raa1* locus as a dominant marker (presence/absence) in all individuals and to map it as a phenotypic marker.

**Table 1:** Segregation analysis of low and high percentage of acylated anthocyanins in carrot F_2_ and F_3_ families 70349, 5392, 5394, and 7095.

Pop. ID	Purple root-source	Generation	No. individuals	Purple vs non-purple segr. ratio	Anthocyanin profile (No. individuals)		Tot.	Exp. seg. ratio	χ^2^
High acylated*	Low acylated*								
5392	P9547	F_3_	150	9:7	68	25	93	3:1	0.17 (P = 0.68)
5394	P9547	F_3_	171	3:1	89	42	131	3:1	3.48 (P = 0.06)
70349	P9547	F_3_	418	9:7	192	47	239	3:1	3.62 (P = 0.06)
7095	P9547 and Ping Ding	F_2_	138	–	36	102	138	3:1	0.13 (P = 0.72)
Total			956				602		

*Low acylated anthocyanins (LAA) include samples with more than 60% of Cy3XGG, and high acylated anthocyanins (HAA) include samples with less than 22% of Cy3XGG.

### Fine Mapping of QTL Conditioning Anthocyanin Acylation

**Figure 2:**
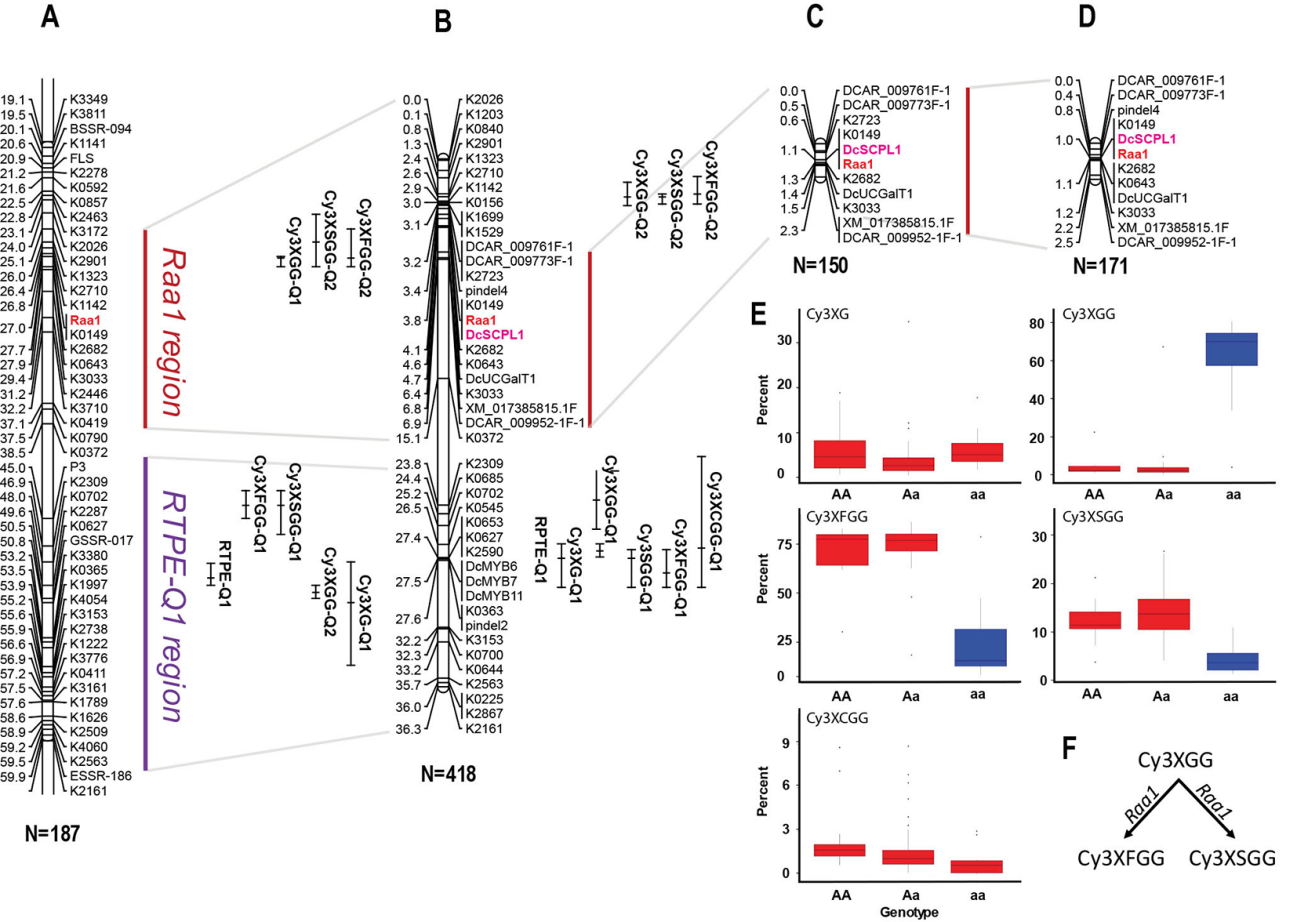
Genetic mapping of anthocyanin pigment traits on chromosome 3 of several carrot populations. **(A)** QTLs for RTPE, root anthocyanin pigments (Cy3XG, Cy3XGG, Cy3XSGG, Cy3XFGG, and Cy3XCGG) and *Raa1*, mapped in the original framework linkage map published by Cavagnaro et al. (2014). The complete linkage group 3 is represented in **Supplementary Figure S3**. **(B)** High-resolution mapping in the RTPE-Q1 and *Raa1* regions using a larger 70349 population. **(C, D)** The *Raa1* locus, conditioning anthocyanin acylation, was mapped in populations 5392 **(C)** and 5394 **(D)** using scores (HAA and LAA) from purple samples. Connecting gray lines indicate the flanking markers of the map region further analyzed in this work. Bars to the right of the linkage groups represent support intervals of the QTLs. The anthocyanin candidate acyltransferase *DcSPL1* is denoted in bold purple letters. Population size is indicated in parenthesis under each linkage group. **(E, F)** Relative content of each cyanidin derivatives based on the allele score at marker K0149. **(F)** Diagram showing the function associated with the *Raa1* locus.

In a previous study by Cavagnaro et al. (2014), we reported on a framework QTL map of population 70349 (*N* = 187), which included two regions in chromosome 3 with co-localized major QTLs for *Raa1* and for root total purple pigmentation (named *RTPE-Q1*) (**Figure 2A** and **Supplementary Figure S3**). The *RTPE-Q1* region harbored QTLs for the cyanidin derivatives and recently, through fine mapping, this region has been associated with the presence of two candidate genes, *DcMYB7* and *DcMYB11*, controlling anthocyanin accumulation in purple roots and petioles, respectively (Iorizzo et al., 2019). The *Raa1* region harbored QTLs for root content of Cy3XGG, Cy3XFGG, and Cy3XSGG. The *Raa1* locus was proposed to regulate the acylation of the cyanidin derivative Cy3XGG (**Figure 2A** and **Supplementary Figure S3**), leading to the accumulation of Cy3XFGG and Cy3XSGG (Cavagnaro et al., 2014). In the present study, HPLC data from 234 additional 70349 population plants, for a total of 418 F_2_ plants, were used along with genotypic data from 39 SNP markers in chromosome 3 including markers covering the *Raa1* region, to construct a linkage map with better resolution of the *Raa1* locus. Consistent with previous findings by Cavagnaro et al. (2014), the resulting 36.3 cM linkage map harbored two regions with co-localized major QTLs for cyanidin derivatives (**Figure 2B**). The region spanning position 23.8 cM to 27.6 cM, harbored co-localized QTLs for all five anthocyanin glycosides and corresponds to the recently fine mapped *RTPE-Q1* region (**Figure 2B** and **Table 2**) (Iorizzo et al., 2019). The region spanning position 2.6 cM to 4.6 cM, harbored co-localized QTLs for Cy3XGG, Cy3XFGG, and Cy3XSGG with LOD scores of 27.3, 16.9, 9.6 and explaining 33.9%, 7.9%, and 6.2% of the observed variation, respectively (**Figure 2B** and **Table 2**). The *Raa1* locus mapped within this QTL region, and co-localized with marker K0149. The *Raa1* map region delimited by the QTL confidence intervals was smaller in the new map, which was constructed using a larger population size (*N* = 418), as compared to the original map (*N* = 187). Together, these 3 QTLs spanned a 3.6 cM region in the framework map (**Figure 2A**) (Cavagnaro et al., 2014), whereas in the new map, co-localized QTLs for three root anthocyanins (Cy3XG, Cy3XSGG, Cy3XFGG) were mapped within a 2 cM region (**Figure 2B** and **Table 2**).

**Table 2:** Summary of QTLs for root total pigment estimate (RTPE) anthocyanin pigments (Cy3XG, Cy3XGG, Cy3XSGG, and Cy3XFGG) and acylation of root anthocyanins (*Raa1*) fine-mapped in carrot chromosome 3 in population 70349.

Trait	QTL ID*	Position (cM)	LOD value	1.5 LOD support interval	Nearest marker	Variation explained (%)
**RPTE**	**RPTE-Q1**	27.5	51	27.0-27.9	DcMYB6-7-11	53
**Cy3XG**	**Cy3XG-Q1**	28.0	29.3	27.0-30	K0627	38.6
**Cy3XGG**	**Cy3XGG-Q1**	24.0	29.2	22.0-26.0	K2309	37.1
	**Cy3XGG-Q2**	4.0	27.3	3.0-4.6	K0149	33.9
**Cy3XSGG**	**Cy3XSGG-Q1**	28.0	48.9	27.4-30.0	K0627	44.5
	**Cy3XSGG-Q2**	4.0	9.6	3.8-4.4	K0149	6.2
**Cy3XFGG**	**Cy3XFGG-Q1**	29.0	67.9	27.4-30.0	K0627	51.2
	**Cy3XFGG-Q2**	3.8	16.9	2.6-4.4	K0149	7.9
**Cy3XCGG**	**Cy3XCGG-Q1**	27.3	30	21.0-30.0	K0627	39.1

*The same IDs were used as in the framework QTL map reported by Cavagnaro et al. (2014).

To further validate the map position of the *Raa1* locus, a total of 11 SNP markers within the *Raa1* region were mapped in populations 5392 and 5394. Linkage maps were obtained for the two mapping populations (**Figures 2C, D**). Analysis of marker order revealed high collinearity across the maps, as can be observed in **Figures 2A–C**, and as indicated by the Spearman rank correlation value of 1.0 obtained for markers order among the maps. *Raa1* was tightly linked (≤ 1.5 cM) to all the SNP markers mapped. In both populations, the marker K0149 completely co-segregated with *Raa1* as observed in population 70349.

ANOVA of the alleles at marker K0149 in all three populations (70349, 5392, and 5394) demonstrated significant (P < 0.01) differences among the different genotypes (*AA*, *Aa*, *aa*) for the relative content of Cy3XGG, Cy3XSGG, and Cy3XFGG (**Figure 2E** and **Supplementary Table S10**). Results (FDR correction, Adjusted P < 0.01) revealed that homozygous dominant “*AA*” genotypes were not significantly different from heterozygous “*Aa*” genotypes for Cy3XGG, Cy3XSGG, and Cy3XFGG, while “*AA*” and “*Aa*” genotypes were significantly different from “*aa*” genotypes (**Supplementary Table S10**). These results suggest that the “*A*” allele is purely non-additive and dominant over the *“a”* allele. The dominant genotypes (*AA* and *Aa*) had high content of the predominant acylated anthocyanins (Cy3XSGG and Cy3XFGG) and less than 22% of Cy3XGG; and the recessive genotypes (*aa*) had low content of these two acylated pigments, and more than 60% of Cy3XGG (**Figure 2E**). These findings suggest that a gene with dominant effect conditions the acylation of anthocyanins, using Cy3XGG as the substrate to produce Cy3XSGG and Cy3XFGG (**Figure 2F**).

In all populations, the closest marker that was not completely segregating with *Raa1* was K2682, which was mapped from *Raa1* at 0.3 cM in population 70349, 0.2 cM in population 5392, and at 0.1 cM in population 5394. Considering the estimated size of the carrot genome (473 Mb) and the length of the integrated linkage map used for assembling the carrot genome sequence (622 cM) (Iorizzo et al., 2016), the average sequence length per map unit is 0.76 Mb/cM. Thus, based on this estimate, the closest marker (K2682) to *Raa1* was located at ~76-228 kb from the trait locus.

### Identification and Analysis of Candidate Genes Controlling Anthocyanin Acylation

**Figure 3:**
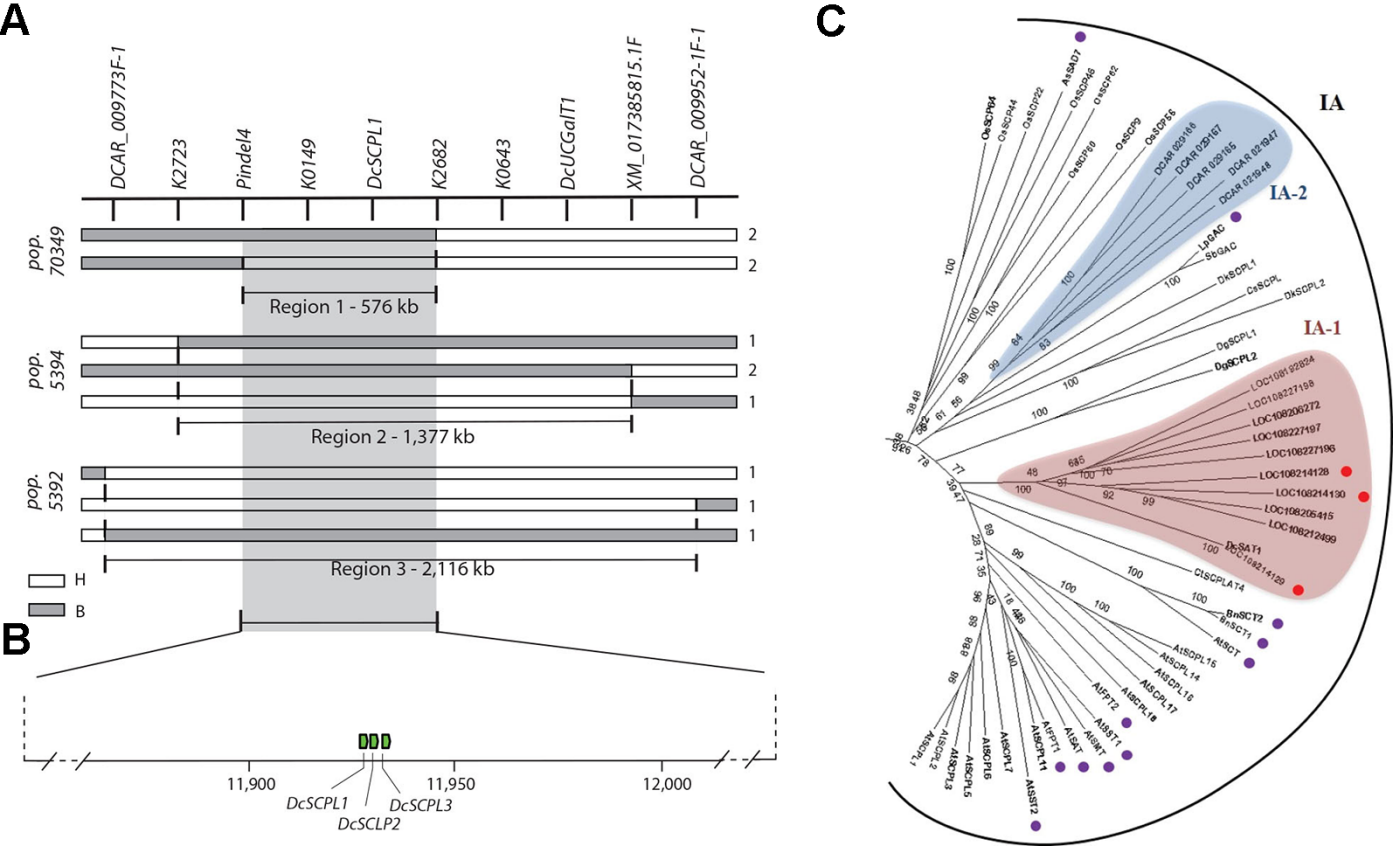
Fine mapping summary and phylogenetic analysis of candidate genes controlling the *Raa1* locus. **(A)** Haplotypes delimiting the genomic regions controlling the *Raa1* locus in population 70349 (region 1), 5394 (region 2), and 5392 (regions 3). Genotyping scores to draw this figure were extracted from **Supplementary Table S11**. The white bars indicate the heterozygous haplotypes (H = *Aa*) and the gray bars indicate the homozygous recessive haplotypes (B = *aa*). Region 1 represent the genomic sequence delimited by the nearest markers flanking the *Raa1* locus across regions 1–3. Numbers on the right side of each bar represent the number of recombinant genotypes for each haplotype. **(B)** Schematic representation of carrot chromosome 3 containing regions 1–3 and three *SCPL acyltransferase* genes (green boxes). The scheme was drawn to scale. **(C)** SCPL phylogenetic clade IA extracted from the neighbor-joining phylogenetic analysis of SCP and SCPL proteins presented in **Supplementary Figure S5**. Bootstrap values are percentage from 1,000 replicates. The scale bar indicates 0.1 substitutions per site. Clades are labeled according to Fraser et al. (2005). Two clusters of carrot SCPL-ATs are highlighted, IA-1 (red) and IA-2 (blue). Protein sequences and complementary information are presented in **Supplementary Tables S4** and **S5**. Red circles indicate SCPL genes located within the *Raa1* locus. Purple circles indicate the following functionally characterized SCPL-ATs. Six SCPL-ATs from *Arabidopsis thaliana*: AtSCT, sinapoylglucose:choline sinapoyltransferase; AtSMT, sinapoylglucose:malate sinapoyltransferase; AtSAT, sinapoylglucose:anthocyanin sinapoyltransferase; AtSST1 and AtSST2, sinapoylglucose:sinapoylglucose sinapoyltransferases; AtFPT1 and AtFPT2, flavonol-phenylacyltransferases. BnSCT1 and BnSCT2, from *Brassica napus*. LpGAC, glucose acyltransferase from *Lycopersicon pennellii*. AsSAD7, sinapoylglucose:sinapoylglucose sinapoyltransferase from *Avena sativa*.

In order to resolve the genomic region containing *Raa1*, analysis of the genotypic scores for the SNP markers flanking *Raa1* was performed to identify recessive (*aa*) to dominant (*A_*) recombination breakpoints associated with changes in the LAA to HAA phenotypes in each of these populations (**Figure 3A** and **Supplementary Table S11**). Alignment of the markers in the *Raa1* region (as defined above) against the carrot genome assembly was used to delimit the corresponding genome sequence. Using this approach, four, four and three linkage blocks associated with recessive (*aa*) to dominant (*A_*) recombinant genotypes, were identified in populations 70349, 5394, and 5392, respectively. Recombination breakpoints were found on each side of *Raa1*, delimiting the genomic region of *Raa1* to 576 Kb in population 70349 (from here on referred to as “region 1”), 1,377 Kb in population 5394 (“region 2”), and 2,116 Kb in population 5392 (“region 3”) (**Figure 3A** and **Supplementary Table S11**). Genomic regions 1–3, harboring *Raa1*, overlapped between markers pindel4 and K2682, which correspond to region 1 (**Figures 3A, B**). These three regions were further analyzed in detail for the identification of candidate genes controlling the *Raa1* genomic region.

Using the gene prediction from the carrot genome assembly DCAR_v2 (Iorizzo et al., 2016) and RefSeq, we found 219 genes in region 3, 68 genes in region 2, and 29 genes in region 1. Except for two UDP-glycosyltransferase genes (*DCAR_009839* and *DCAR_009912*), none of the predicted genes located in regions 1–3 correspond to previously annotated candidate genes involved in the anthocyanin or the flavonoid biosynthetic pathways (Iorizzo et al., 2016). Both UDP-glycosyltransferase genes are located outside of region 1 (**Figures 2A–D** and **3A**) and *DCAR_009912* (*DcUCGalT1*) has been recently shown to catalyze the formation of Cy3G (Xu et al., 2016). However, none of the carrot genes previously annotated as members of the anthocyanin or the flavonoid biosynthetic pathways include acyltransferases (Iorizzo et al., 2016). To establish the first list of potential acyltransferases that could catalyze anthocyanin acylation in carrot, we performed an orthologous and phylogenetic analysis using all predicted carrot genes (DCAR_v2 and RefSeq predictions) and 83 sequences of characterized and putative flavonoid-related acyltransferases (from the SCPL and BAHD families), and 39 sequences of hydrolytic serine carboxypeptidases (from the SCP family) (**Supplementary Table S4**).

In total, 87 carrot genes were identified through orthologous analysis as potentially coding for an acyltransferase (**Supplementary Table S5**). The phylogenetic analysis revealed that 43 carrot genes clustered with known BAHD acyltransferases coding genes, but none of them were located within “regions 1–3” of the *Raa1* locus (**Supplementary Figure S4**). Phylogenetic analysis of the SCP/SCPL family revealed two clades previously defined by Mugford et al. (2009): clade I contains the SCPLs and clade II the SCPs (**Supplementary Figure S5**). Functionally characterized SCPL-ATs are all grouped into clade IA, whereas clade IB corresponds to a subgroup of potentially divergent SCPLs, from both monocot and dicot species (**Supplementary Figure S5**). All carrot SCPL-ATs are clustered into two distinct subgroups: IA-1 and IA-2 (**Figure 3C**). Clade IA-1 contains 10 carrot SCPL-ATs, including three that are encoded by a tandem cluster of genes located within the 576 kb fine mapped region controlling the *Raa1* locus: *LOC108214129* (*DcSCPL1*), *LOC108214128* (*DcSCPL2*), and *LOC108214130* (*DcSCPL3*) (**Figures 3B, C**). Interestingly, *DcSAT1*, a putative SCPL-AT coding gene that has been recently identified as a potential target of *DcMYB7*, clustered with *DcSCLP1* (**Figure 3C**) (Xu et al., 2019). The *DcSCPL1* coding sequence is about 1.4 kb and is composed of 14 exons. Alignment of the *DcSAT1* mRNA (GeneBank ID: MK572824) and its promoter region (GeneBank ID: MK572826.1) against the carrot genome, indicated that these sequences correspond to *DcSCPL1* and its promoter region, respectively. *DcSAT1* and *DcSCPL1* protein sequences are identical, demonstrating that these two genes represent the same locus in the carrot genome and from here on we will refer to *DcSCPL1* as a synonymous of *DcSAT1*.

DcSCPL1, DcSCPL2, and DcSCPL3 protein sequences are over 59% pairwise identical between each other; DcSCPL2 and DcSCPL3 being the most similar with 68% homology. Sequence analysis shows that all three genes possess the Peptidase_S10 family domain (PF00450) in C-terminal (**Supplementary Figure S6**). However, DcSCPL3 is missing 38 amino acids within the domain, including one of the amino acids from the conserved catalytic triad, essential for SCP/SCPL function (Mugford and Milkowski, 2012; Bontpart et al., 2018). A search for the N-terminal secretory signal peptide, a motif characteristic of SCPL acyltransferases which allow the transport of the protein to the vacuole, revealed its potential presence in DcSCPL1 and DCSCPL3 but not in DcSCPL2 (**Supplementary Figure S6C**) (Bontpart et al., 2015). DcSCPL1 is the only SCPL-AT being encode by a gene located in the fine mapped region and predicted to possess the required domain/motifs to be functional. Mapping of *DcSCPL1* in populations 70349, 5392, and 5394 confirmed its co-localization with *Raa1* (**Figures 2B–D**).

### Expression Analysis of the *SCPL Acyltransferase* Genes in *Raa1*

**Figure 4:**
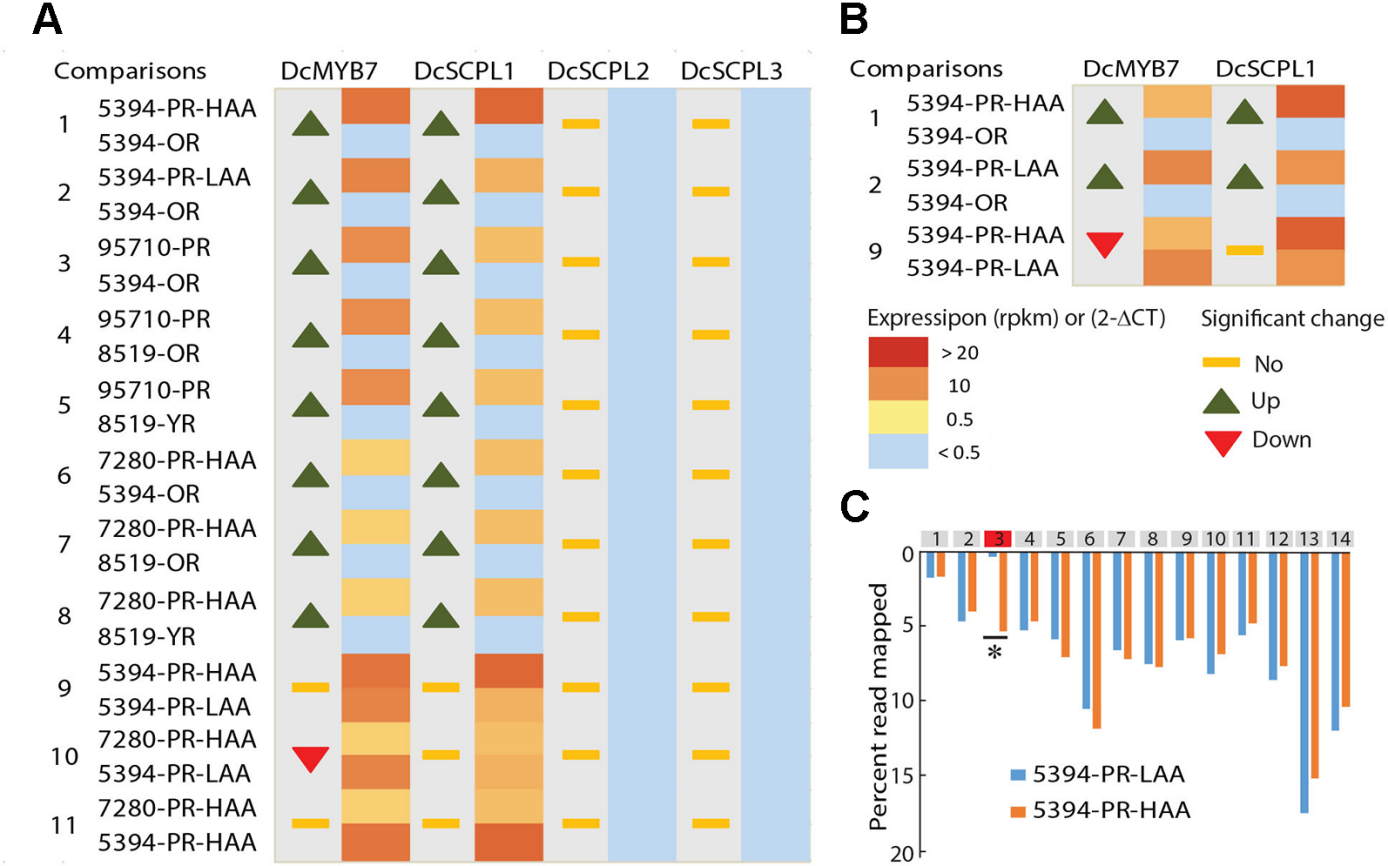
Comparative transcriptome analysis of candidate genes potentially involved in anthocyanin acylation of carrot roots. **(A)** RNA-Seq data from seven different carrot root tissues and across 11 comparisons (**Supplementary Tables S6**–**S7**). The heatmaps represent the expression level from yellow (< 0.5 RPKM) to red (> 20 RPKM). Samples with a normalized read level <0.5 RPKM, were considered non-expressed and are labeled in blue. For each comparison, statistically significant fold changes (Log FC) in expression between tissues are indicated by an up or down arrow. **(B)** RT-qPCR results in 5394 root tissues, from three biological replicates. Heatmaps represent the relative expression of *DcSCPL1* and *DcMYB7* using the 2^-ΔCT^ method. For each comparison, statistically significant fold changes through ANOVA analysis (P < 0.01) are indicated by an up or down arrow. **(C)** Distribution of the reads mapping to each exon of *DcSCPL1*, in 5394-PR-HAA and 5394-PR-LAA plants. All exons are represented by gray boxes except exon-3 that is represented in red. The quantification of the reads mapping each exon is expressed in percentage of the total number of reads mapping *DcSCPL1*. Statistically different expression of each exon (*) between HAA and LAA plants were tested using a negative binomial test with RPKM value (Log2FC > 1 and FDR ≤ 0.5). The statistical results of each exon are presented in **Supplementary Table S12**.

Genome wide quantitative transcriptome analysis was performed in selected samples representing purple pigmented (HAA and LAA) and non-pigmented root tissues. These included four purple-rooted carrots (5394-PR-HAA, 5394-PR-LAA, 7280-PR-HAA, and 95710-PR), two orange-rooted carrots (5394-OR and 8519-OR), and one yellow-rooted carrot (8519-YR) (Bannoud et al., 2019). After cleaning low quality reads, an average of 35 million high-quality reads per biological replicate were retained for further downstream analysis (**Supplementary Table S6**). In total, 11 pairwise comparisons of purple *versus* non-purple root tissues (comparison 1–8) and HAA *versus* LAA purple tissues (comparison 9–11) were performed to identify DEGs from the 576 kb genomic region harboring the *Raa1* locus (**Figure 4A** and **Supplementary Table S7**).

In all samples, both *DcSCPL2* and *DcSCPL3* were detected below a measurable threshold of 0.5 Reads Per Kilobase Million (RPKM) and were considered not expressed. On the other hand, *DcSCPL1* appears to be consistently expressed in all purple-rooted lines (HAA and LAA), reaching over 22 RPKM in 5394-PR-HAA samples, and not expressed in orange or yellow roots (comparisons 1–8, **Figure 4A**). The purple-specific expression of *DcSCPL1* highly correlates with the expression of *DcMYB7,* which regulates anthocyanin biosynthesis in carrot root and was recently proposed to directly control the expression of *DcSCPL1* (Xu et al., 2019)*. DcUCGalT1*, also involved in anthocyanin biosynthesis (Xu et al., 2016), had an expression profile similar to *DcSCLP1*, suggesting a possible co-regulation to coordinate anthocyanin LBGs activity (**Supplementary Table S7**). These results indicate that *DcSCLP1* is the only acyltransferase gene located in the fine-mapped region that is consistently expressed in purple samples (LAA and HAA), and support the hypothesis proposed by Xu et al. (2019) that it is regulated by *DcMYB7*.

To investigate if a correlation exists between *DcSCPL1* expression level and the low *versus* high acylation profile of anthocyanins in the carrot root, we compared our RNA-Seq data from 5394-PR-HAA and 7280-PR-HAA to 5394-PR-LAA root samples (comparisons 9–10, **Figure 4A**). No significant differences in expression were observed for each of the two comparisons. This indicates that the level of expression of *DcSCPL1* does not have an additive effect and suggests that the mutation affecting anthocyanin acylation in population 5394 LAA plants does not affect the regulation of *DcSCPL1* expression level.

To validate the RNA-Seq-based gene expression profiles, the expression levels of *DcMYB7* and *DcSCPL1* were examined by RT-qPCR analysis using mRNA extracted from 5394-OR, 5394-PR-LAA, and 5394-PR-HAA root tissues (**Figure 4B**). Both genes were up-regulated in purple *versus* non-purple root comparisons (comparisons 1–2), consistent with the RNA-Seq data. As expected, when comparing 5394-PR-HAA with 5394-PR-LAA root tissues, *DcSCPL1* expression was not significantly different (comparison 9). However, *DcMYB7* was slightly down-regulated in HAA root tissue (comparison 9).

The absence of variation of *DcSCPL1* expression between HAA *versus* LAA content, suggests that the control of anthocyanin acylation in the mapping populations could be due to a mutation affecting the structure rather than the accumulation of *DcSCPL1* transcripts. Analysis of the distribution of all the reads mapped to *DcSCPL1*, across its 14 exons, revealed a similar distribution pattern between HAA and LAA homozygous plants (according to the haplotype), excepted for the third predicted exon (**Figure 4C** and **Supplementary Table S12**). In LAA plants, only 0.3% of the reads map to exon-3 as opposed to 5.3% in HAA plants, suggesting that an alternative *DcSCPL1* transcript, lacking part or all of the exon-3, is expressed in 5394 LAA plants. These results suggest that a mutation, causing an alteration of *DcSCPL1* transcripts, could be responsible for the lower acylation activity observed in the LAA samples.

### Identification of a *DcSCPL1* Mutation in Population 5394 LAA Plants

**Figure 5:**
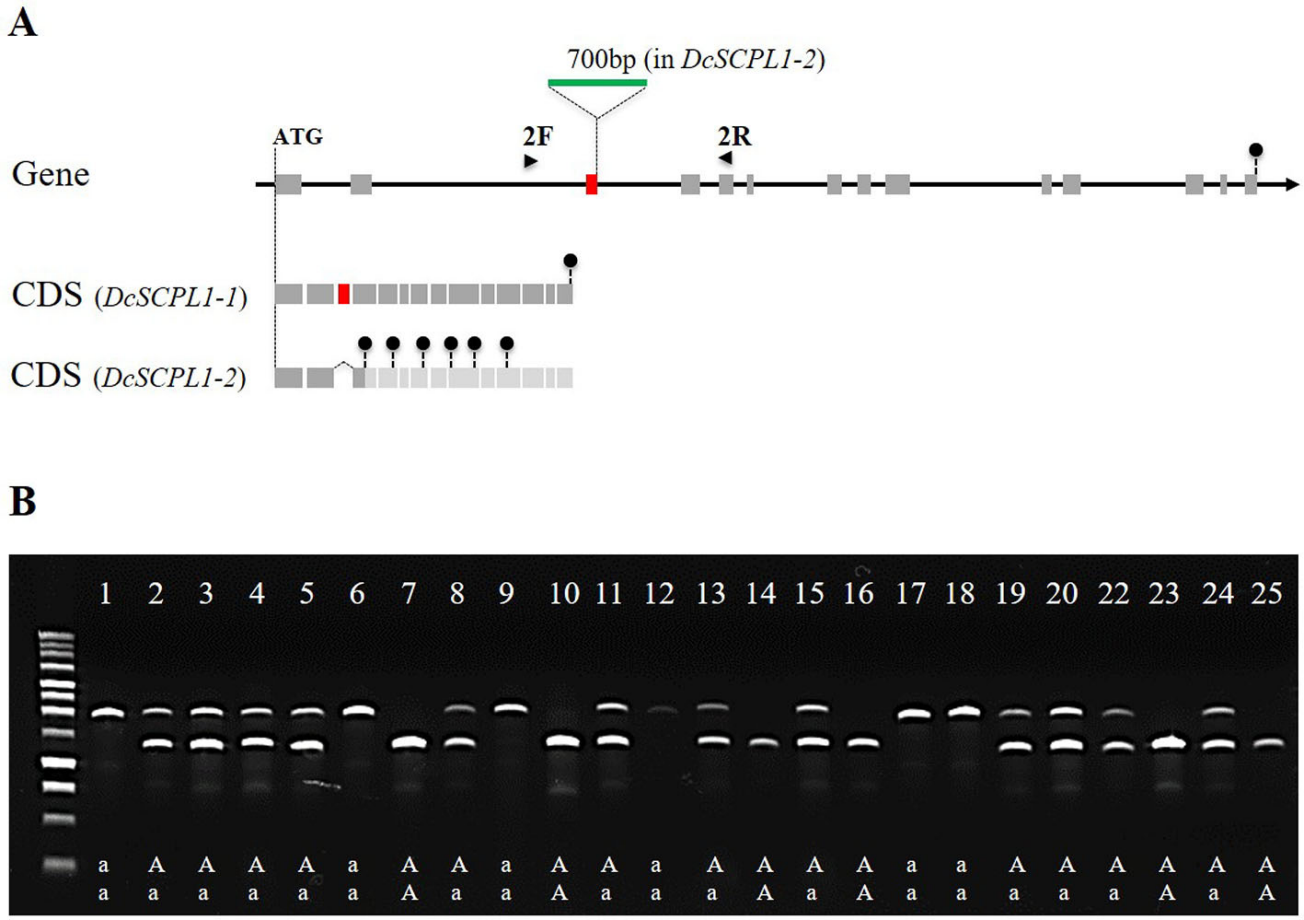
Polymorphism of the *DcSCPL1* locus in the 5394 population. **(A)** Structure of the *DcSCPL1* full-length gene and coding sequences (CDS) of the dominant (“*AA*”, *DcSCPL1-1*) and recessive (“*aa*”, *DcSCPL1-2*) alleles. *DcSCPL1-2* contains a 700 bp insertion (green) at the junction between the 3^rd^ exon (red) and its following intron, resulting in an alternative transcript, a shift of the open reading frame and premature stop codons (black circles). Light gray exons indicate that the predicted sequence can no longer be translated due to the presence of early stop codons. 2F and 2R indicate the position of the forward and reverse primers used for genotyping. **(B)** Genotyping by PCR of the *DcSCPL1* locus in 25 plants from the 5394 population. Haplotypes (“*aa*”, “*Aa*”, or “*AA*”) are indicated based on the *DcSCPL1* SNP marker genotyped in these individuals. Expected amplicons length: 1200 bp (without insertion) or 1900 bp (with insertion).

To further investigate mutations in *DcSCPL1* mRNA, we sequenced it in the segregating populations 5392 and 5394. Based on the haplotype, we used three homozygous lines for each HAA and LAA phenotypes and identified a different cDNA sequence for each genetic background (**Supplementary Figure S7**). Sequence comparison revealed multiple polymorphisms. *DcSCPL1-1* allele, in HAA plants, is 100% identical to the sequenced carrot DH1 predicted mRNA. The *DcSCPL1-2* allele, in LAA plants, contains eight single nucleotide polymorphisms (SNPs) and a 77 bp deletion corresponding to the entire third predicted exon. This deletion creates a shift of the open reading frame and the presence of premature translation termination codons, which should result in the production of a shorter, non-functional, protein (**Supplementary Figure S7**). To further assess the origin of this deletion we sequenced the genomic region surrounding exon-3 in *DcSCPL1*, in both LAA and HAA plants, and identified a 700 bp insertion located exactly at the exon-3/intron-3 junction (**Figure 5A** and **Supplementary Figure S8**). This insertion changes the 5’ splice site, located at the 5’end of intron-3, from “GUGuGU” to “GUAgGg” [lower cases indicate nucleotides diverging from the established 5’ splice site consensus (Galej, 2018)] possibly affecting the splicing of the pre-mRNA and explaining the deletion of exon-3 in *DcSCPL1-2* transcripts.

To verify that this mutation correlates with the LAA phenotype in the 5394 mapping population, we used a pair of primers surrounding the insertion to screen 25 plants for the presence of each of the *DcSCPL1* alleles (**Figure 5A**). The detection of *DcSCPL1-1* allele produced a 1200 bp amplicon (lower band), while the detection of *DcSCPL1-2* allele produced a 1900 bp amplicon (upper band). All the LAA plants (1, 6, 9, 12, 17, and 18) were genotyped as homozygous for the *DcSCPL1-2* allele, while at least one *DcSCPL1-1* allele was detected in all the HAA plants (**Figure 5B**). For all individuals, the detection of *DcSCPL1* alleles perfectly correlated with the haplotypes from the SNP marker genotyped.

Overall our results indicate that an insertion in the *DcSCLP1* locus of 5394 LAA plants is probably the cause of the truncation of *DcSCPL1* transcripts, which likely translate into a non-functional proteins to cause the reduced acylation of Cy3XGG and the LAA phenotype associated with the *Raa1* locus.

## Discussion

### *DcSCPL*1 is the Best Candidate Gene Controlling Cy3XGG Acylation in Carrot

To date, multiple QTLs controlling the accumulation of different forms of cyanindin derivatives including the acylated forms Cy3XFGG, Cy3XSGG, and Cy3XCGG, in carrot root have been detected and mapped. Cavagnaro et al. (2014) identified six QTLs associated with acylated anthocyanins, and described a major locus named *Raa1*, that control the accumulation of high *versus* low acylated anthocyanins content in the storage root. Recently, Bannoud et al. (2019) identified over 20 QTLs associated with acylated anthocyanins in the carrot root phloem and xylem tissues. However, the populations used by Bannoud et al. (2019) did not segregate for the *Raa1* locus and accumulated large amount of acylated anthocyanin which resembles the HAA phenotype described in the present study.

Here we report on the first study in carrot that integrates linkage mapping and candidate gene identification for acylated anthocyanin accumulation in the carrot root. We delimited the region of *Raa1* to a 576 Kb region of the long arm of chromosome 3. Allele-phenotype interaction analysis indicated that the gene controlling the *Raa1* locus has a dominant effect, with HAA being dominant over LAA. A comprehensive analysis to predict acyltransferase genes, through orthologous and phylogenetic analysis, identified a cluster of three SCPL-ATs genes (*DcSCPL1*, *DcSCPL2*, and *DcSCPL3*) in this region. Sequence analysis revealed that *DcSCPL1* corresponds to *DcSAT1*, a putative acyltransferase recently identified as a potential target of *DcMYB7* (Xu et al., 2019). Protein sequence analysis indicated that only *DcSCPL1* contains all conserved domains required for the acyltransferase activity, while *DcSCPL2* secretory pathway toward the vacuole may be compromised by the absence of a functional N-terminal signal peptide and *DcSCPL3* activity is likely to be impaired by the partial deletion of its catalytic domain. Although we cannot rule out that *DcSCPL2* and *DcSCPL3* protein sequences, extracted from the DH reference genome, may be different in the populations studied here, *DcSCPL1* sequence is identical between DH and HAA plants, suggesting that the same is true for all three clustered genes. Sequence polymorphism between the three genes also extend to their promoter regions for which they share no significant homology (data not shown). Comparative transcriptome (RNA-Seq) and gene expression (RT-qPCR) analyses demonstrated that *DcSCPL2* and *DcSCPL3* were not expressed in the tested samples, suggesting that both genes do not possess the necessary cis-regulatory element(s) to control their promoter activity. *DcSCPL1* is the only predicted acyltransferase gene located in the *Raa1* region to be expressed and up-regulated in all the purple (*vs.* non-purple) root samples, but was not differentially expressed between LAA and HAA carrot roots. Sequence and transcriptome data analysis revealed that an insertion within *DcSCPL1* coding sequence is associated with the LAA genotype and is likely causing the lower anthocyanin acylation observed in LAA plants. Overall, our results demonstrated that *DcSCPL1* represent the best candidate gene controlling the *Raa1* locus.

LAA plants of population 5394, which are homozygous for the *DcSCPL1-2* recessive allele, still produce about 4 to 36% of combined amount of Cy3XSGG and Cy3XFGG. According to our RNA-Seq data presented in **Figure 4C**, 0.3% of *DcSCPL1* reads are mapping to the 3^rd^ exon in *DcSCPL1-2* (recessive allele), as oppose to 5.3% in *DcSCPL1-1* (dominant allele), homozygous plants. This result suggests that a residual low fraction of the dominant allele is transcribed in the *DcSCPL1* recessive LAA plants. We hypothesized that the 700 bp insertion in *DcSCPL1-2*, located at the exon-3/intron-3 splice junction, does not always disrupt the splicing of the 3^rd^ exon, therefore generating, in some cases, a full length *DcSCPL1* transcript. Using primers specific to each of the two *DcSCPL1* alleles, we were able to confirm by RT-qPCR the detection of traces of full length *DcSCPL1* transcripts in 5394-LR-LAA plants (**Supplementary Figure S9**). This alternative splicing of *DcSCPL1-2* transcripts could be responsible for the production of low levels of functional DcSCPL1 proteins, which likely accounts for at least part of the biosynthesis of the remaining Cy3XSGG and Cy3XFGG detected in LAA plants.

Besides of *DcSCPL1*, *DcSCPL2*, and *DcSCPL3*, our phylogenetic analysis revealed 15 others *DcSCPL*s (**Supplementary Figure S5**). Only three of them, LOC108192824, LOC108227198, and LOC108205415, which clustered with *DcSCPL1* in clade IA-1, possess the predicted functional SCPL domain/motifs and are encoded by genes expressed above the detection threshold level in all purple roots (**Supplementary Table S7** and **Figure S10**). These three proteins represent obvious candidates to investigate the genetic mechanism controlling the accumulation of low levels of acylated anthocyanins in LAA plants, and/or the preferential use of certain acyl donors. For example, these three *DcSCPL*s are also candidates for the acylation of Cy3XGG with p-coumaroyl to produce Cy3XCGG.

### *DcSCPL1* Act in a Substrate Specific Manner

Our study suggests that *DcSCPL1* regulates acylation in a substrate preferential manner. The reduced level of the acylated anthocyanins Cy3XSGG and Cy3XFGG in the LAA individuals was negatively correlated with the increased level of Cy3XGG, strongly indicating that DcSCPL1 uses Cy3XGG as substrate. To the best of our knowledge, no study in carrot has reported the detection of Cy3XG acylated forms, which could be an alternative substrate to Cy3XGG, used by *DcSCPL1*. Closely related AtSCPL-ATs were shown to specifically react with different acyl acceptors, suggesting a high substrate specificity for SCPLs varying by a relatively small subset of amino acids (Fraser et al., 2007). These observations further support that *DcSCPL1* catalyzes specifically the acylation of Cy3XGG in a substrate-specific fashion.

*DcSCPL1* protein sequence shares over 61% pairwise sequence similarity with 5 characterized *Arabidopsis* SCPL acyltransferases (SMT, SCT, SST1, SST2, and SAT), that all use specifically sinapoylglucose as the acyl donor, suggesting a similar activity in carrot (Mugford and Milkowski, 2012). Recently, Xu et al. (2019) demonstrated that the overexpression of *DcMYB7* in orange carrot “Kurodagosun” promotes *DcSAT1* (synonymous of *DcSCPL1*) expression and the biosynthesis of Cy3XSGG anthocyanin. These results suggest that DcSCPL1 could preferentially use sinapoylglucose, rather than feruloylglucose or coumaroylglucose, as acyl donor to catalyze the transesterification of Cy3XGG (Xu et al., 2019). In our study, the inverse correlation between the levels of Cy3XSGG and Cy3XFGG, in the HAA dominant genotypes, indicates that DcSCPL1 could use both sinapoylglucose and feruloylglucose as acyl donors to catalyze the acylation of Cy3XGG into Cy3XSGG and Cy3XFGG. Our data also indicated that Cy3XFGG is always present in larger amount than Cy3XSGG (**Figures 1B, C** and **2E**), suggesting that DcSCPL1 may preferentially use feruloylglucose, rather than sinpoylglucose, as acyl donor in the carrot genotypes used in this study. Cy3XCGG is also an important form of acylated anthocyanin in carrot, that can be present, in some cultivars, at a similar or higher level as Cy3XSGG (Montilla et al., 2011; Assous et al., 2014). In our mapping population, Cy3XCGG is present at up to 5% of relative concentration, and its biosynthesis is not significantly correlated with *DcSCPL1* alleles (**Figure 2E**). Considering the potential high substrate specificity of acyltransferases, other genes, such as the *SCPL-AT* candidates mentioned previously, could be involved in the biosynthesis of the different types of acylated anthocyanins described in carrot.

Alternatively, the different ratios among the acylated forms of anthocyanin could be due to the presence of differential amounts of acyl donors, feruloylglucose and/or sinapoylglucose, in the mapping populations used in our study. This could favor the synthesis of Cy3XFGG over Cy3XSGG, and vice versa, by *DcSCPL1*. This hypothesis could also explain the different ratio of anthocyanin acylated forms detected in other studies and genetic backgrounds. Investigating the functionality of *DcSCPL1* and other *DcSCPL*s genes identified in this study, through *in vitro* or *in vivo* experiments, would need to be performed in order to clearly define their enzymatic function. For example, their activity could be assessed in different purple genetic backgrounds, such as Antonina and Deep Purple *versus* Beta Sweet and Purple Haze, that contain different ratios of those two forms of acylated cyanidin (Montilla et al., 2011; Algarra et al., 2014).

### Landscape of Flavonoid Related BAHD Acyltransferases in Carrot as Candidate for Anthocyanin Acylation in Carrot

Many BAHD acyltransferases are involved in the production of phenolic secondary metabolites including anthocyanin acylation (Bontpart et al., 2015). Gt5AT, one of the founding members of the BAHD family, uses p-coumaroyl-CoA acid as substrate to acylate anthocyanins (Fujiwara et al., 1997). Previous phylogenetic analysis identified 5 BAHD acyltransferases clades (D’Auria, 2006; Tuominen et al., 2011), with clade I containing all of the characterized BAHDs catalyzing anthocyanin modification, except Ss5MAT2. Accordingly, our phylogenetic analysis using only flavonoid BAHD acyltransferases revealed two clades: clade I containing all the previously identified clade I BAHDs, and clade II containing Ss5MAT2 (**Supplementary Figure S4**). Most of the characterized anthocyanin BAHDs acyltransferases use malonyl-CoA as acyl donor (MAT enzymes) while others use hydroxycinnamoyl-CoA (AT enzymes) (**Supplementary Figure S4**) (Bontpart et al., 2015). So far, anthocyanin BAHDs acyltransferases have been shown to use p-coumaroyl-CoA, p-feruloyl-CoA or p-caffeoyl-CoA, but not p-sinapoyl-CoA, as acyl donor (Fujiwara et al., 1998; Yonekura-Sakakibara et al., 2000; Luo et al., 2007; Rinaldo et al., 2015). This suggests that BAHDs are good candidates to catalyze the formation of Cy3XCGG and Cy3XFGG, but not Cy3XSGG, in carrot.

Because of the versatility of BAHDs, it is difficult to predict the substrate specificity of an enzyme based on its sequence (Luo et al., 2007). Nonetheless, specific structural motifs corresponding to clade I and anthocyanin BAHD acyltransferases have been proposed (D’Auria, 2006; Tuominen et al., 2011). Out of 43 DcBAHDs sequences identified in our phylogenetic analysis, 35 possess both the HxxxD and the DFGWG motifs characteristic of functional BAHDs (**Supplementary Figures S11** and **S12**). Eleven of them possess (allowing one amino acid variation) the clade I specific PLTFFD motif and the anthocyanin acyltransferase specific YFGNC motif, which is thought to be involved in acyl donor recognition (D’Auria, 2006; Unno et al., 2007; Tuominen et al., 2011) (**Supplementary Figures S11** and **S12**). Our RNA-Seq data show that only three of them, encoded by: *DCAR_030734*, *DCAR_021223*, and *LOC108224500*, are being expressed at more than 1 RPKM in all of the purple root samples. Interestingly, *DCAR_021223* and *LOC108224500* are found in two tandem clusters of highly similar genes, located within the QTL associated with Cy3XSGG accumulation in the root (Cavagnaro et al., 2014), making them candidates to participate in anthocyanin modification. Many anthocyanin acyltransferases are organized in tandem clusters, which is consistent with the hypothesis of a recent evolution (Fraser et al., 2007; Stehle et al., 2009). *DCAR_030734*, on the other hand, is the only one not clustering with other DcBAHDs in clade I, suggesting an older evolution and a conserved function (**Supplementary Figure S4**).

### Coordination of the Anthocyanin LBGs Expression by DcMYB7

*DcMYB7* expression has consistently been associated with anthocyanin accumulation (Bannoud et al., 2019; Iorizzo et al., 2019; Xu et al., 2019). Recently, Xu et al. (2019) showed evidences that *DcMYB7* could activate the expression of its *DcbHLH3* partner, as well as the glycosylation and acylation of anthocyanins by directly activating *DcUCGXT1* and *DcSAT1* (synonymous of *DcSCPL1*). The large set of RNA-Seq data available in this study, derived from different genetic backgrounds, provide us the first opportunity to identify genes that are co-expressed with *DcMYB7*. In agreement with Xu et al. (2019), our data demonstrated that all three potential *DcMYB7* target genes (*DcbHLH3*, *DcUCGXT1*, *DcSCPL1*) are constantly co-expressed with *DcMYB7* in purple roots and not expressed in orange or yellow roots (**Supplementary Figure S7**). Additionally, the gene encoding for *DcUCGalT1*, which was recently shown to catalyze the formation of Cy3G (Xu et al., 2016) and which is located within region 2 of the *Raa1* locus, follows the same co-expression pattern (**Supplementary Figure S7**), suggesting that it could be also directly regulated by *DcMYB7*. Neither *DcSCPL2* nor *DcSCPL3*, co-localizing with *DcSCPL1* in the *Raa1* locus, are co-expressed with *DcMYB7*. These results leave little doubt that *DcMYB7* function as master regulator to coordinate anthocyanin biosynthesis in carrot root across cultivars, and therefore reinforce our hypothesis that *DcSCPL1* is an important structural gene of the anthocyanin acylation pathway.

Considering the substrate specificity and the diversity of acylated anthocyanins that can be formed, multiple acyltransferases are likely involved in the control of anthocyanin acylation in carrot, and their regulation may be more complex than just a transcriptional activation by a master regulator like *DcMYB7*. Changes in environmental conditions, such as temperature during the crop cycle, can influence anthocyanin composition, and particularly the proportion of acylated anthocyanins, in other plant species (Tarara et al., 2008; de Rosas et al., 2017). Although the influence of environmental factors on carrot anthocyanin composition has not been studied to date, caution is advised when inferring on a carrot *DcSCPL1* genotype based on its anthocyanin composition, especially when comparing data from different studies or among field-grown carrots from different regions. In the present study, the plants from each segregating population were grown under the same environmental conditions, and consequently the observed variation in “% acylated anthocyanins” are not likely attributed to differences in environmental influence but rather to the genetic variation found in the *DcSCPL1* gene.

The biosynthesis of particular types of anthocyanins with unique decoration patterns could be triggered in response to distinct abiotic stress and it is likely that acyltransferase coding genes are subject to multiple layers of regulation (Kovinich et al., 2014). Alternative splicing is emerging as an important regulatory mechanism in plant adaptation to environmental stress (Laloum et al., 2018; Huertas et al., 2019). It is possible that the splicing of *DcSCPL1* transcript is not only affected by the 700 bp insertion in *DcSCPL1-2* but may be controlled by a larger regulatory mechanism. Could LBGs controlling anthocyanin decoration be more susceptible to alternative splicing as a way for the plant to increase enzyme diversity and consequently the pool of anthocyanin produced in response to abiotic stress? Functional studies through enzymatic assays and/or transgenic approaches will be required to precisely characterize the function and understand the regulations of acyltransferases involved in the processing of anthocyanin in carrot.

## Data Availability Statement

The RNA-Seq datasets analyzed for this study can be found in the GenBank Short Read Archive under the umbrella project number PRJNA484382.

## Author Contributions

MI designed the study. PS developed and grew the plant populations. DS and PC performed HPLC analysis. MI and PC genotyped and mapped *Raa1*, *DcSCPL1*, *DcUCGXT1*, and *DcMYB7* in the 70349 background. YZ performed KASPar genotyping analysis. HB performed all bioinformatics analyses. JC performed real time PCR, and candidate gene analysis. MM performed the pigmentation and inheritance analysis. MI and JC interpreted results. MI, JC, and HB drafted sections of the manuscript and prepared figures and tables. PS, MM, YZ, PC, and DS critically revised the manuscript. MI and JC prepared the final version of the paper. All authors read, reviewed, and approved the manuscript.

## Funding

MI, HB, YZ, and JC were supported by the United States Department of Agriculture National Institute of Food and Agriculture, Hatch project 1008691. This project was also supported by the National Institute of Food and Agriculture, United States Department of Agriculture, under award number 2016-51181-25400, project “Identifying phenotypes, markers, and genes in carrot germplasm to deliver improved carrots to growers and consumers.” PC was supported by the “Agencia Nacional de Promoción Científica y Tecnológica” through project “PICT-2015-1625”.

## Conflict of Interest

The authors declare that the research was conducted in the absence of any commercial or financial relationships that could be construed as a potential conflict of interest.
