# Supplementary material for: Identification of an SCPL Gene Controlling Anthocyanin Acylation in Carrot (Daucus carota L.) Root
Source: Front Plant Sci. 2020 Jan 31;10:1770. doi: 10.3389/fpls.2019.01770 (PMC7005140; doi:10.3389/fpls.2019.01770)
Supplement: Supplementary file 1 [file DataSheet_1.pdf]

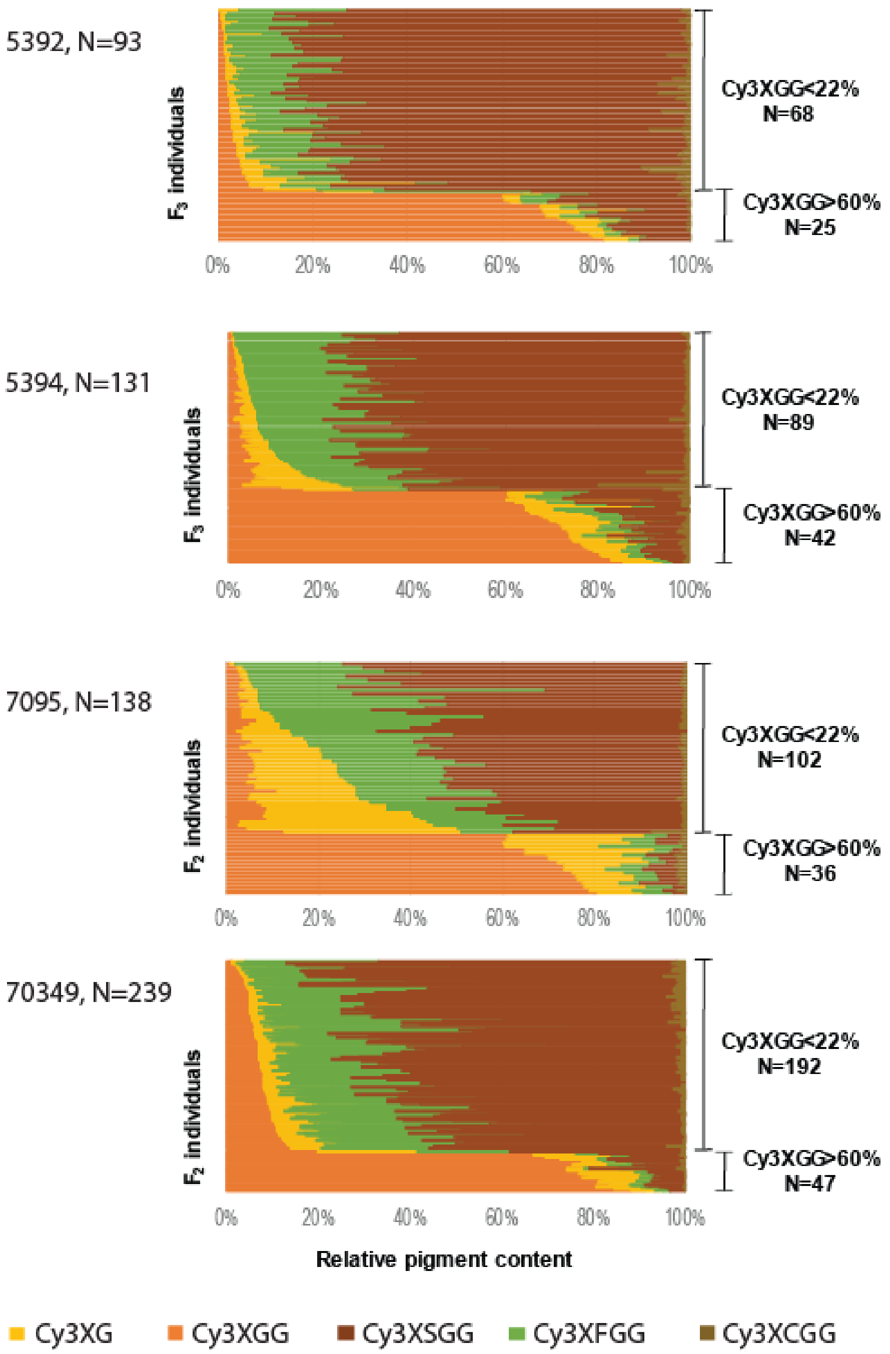

Supplementary Figure S1. Relative content (%) of five cyanidin derivatives (Cy3XG, Cy3XGG, Cy3XSGG, Cy3XFGG, and Cy3XCGG) in purple-rooted individuals of 5392, 5394, 7095 and 70349 populations. Only plants with purple root were evaluated by HPLC anthocyanin analysis. Plants with low percentage of Cy3XGG (< 22%) are considered HAA and plants with high percentage of Cy3XGG (> 60%) are considered LAA.

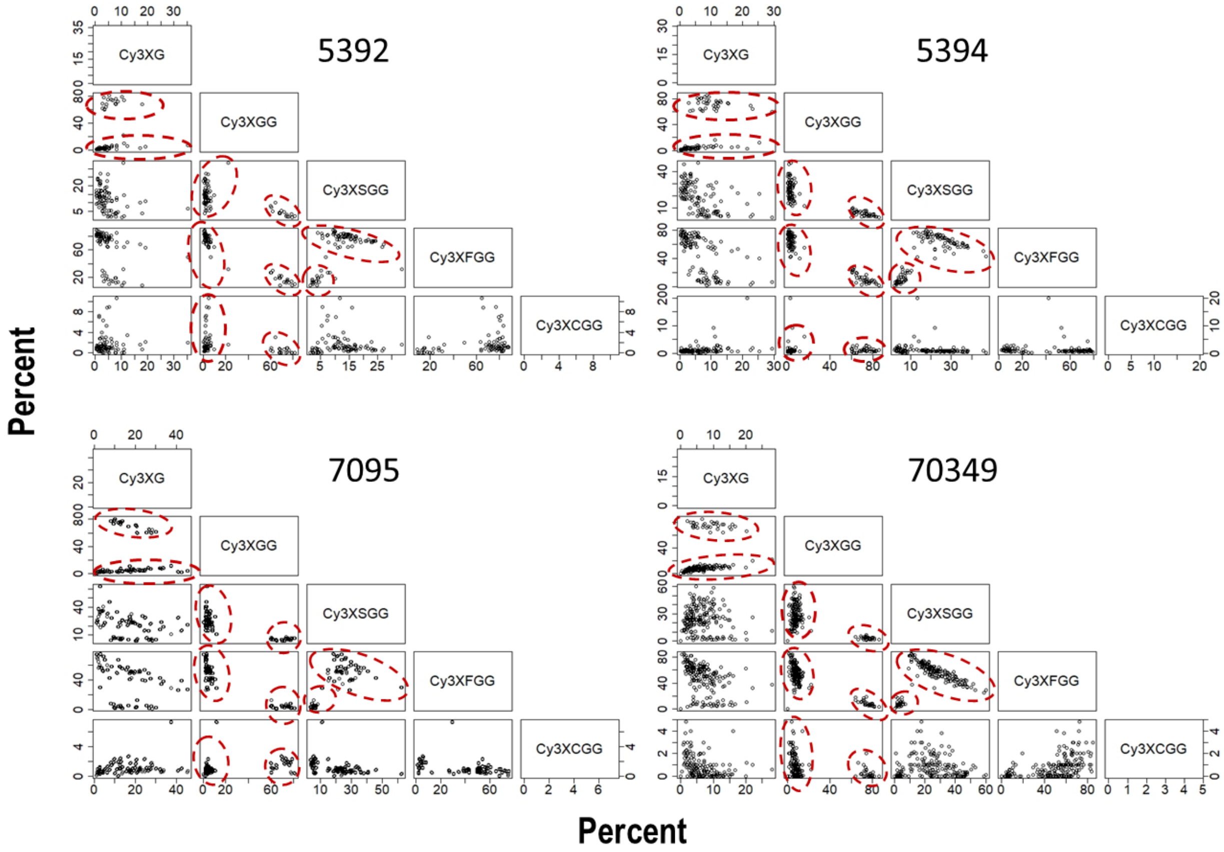

Supplementary Figure S2. Scatter plot analysis for pairwise correlations among the five cyanidin derivatives in populations 5392, 5394, 7095 and 70349. Only plants with purple roots were evaluated by HPLC anthocyanin analysis. Circles indicate individuals with less than 22% and more than 60% of Cy3XGG.

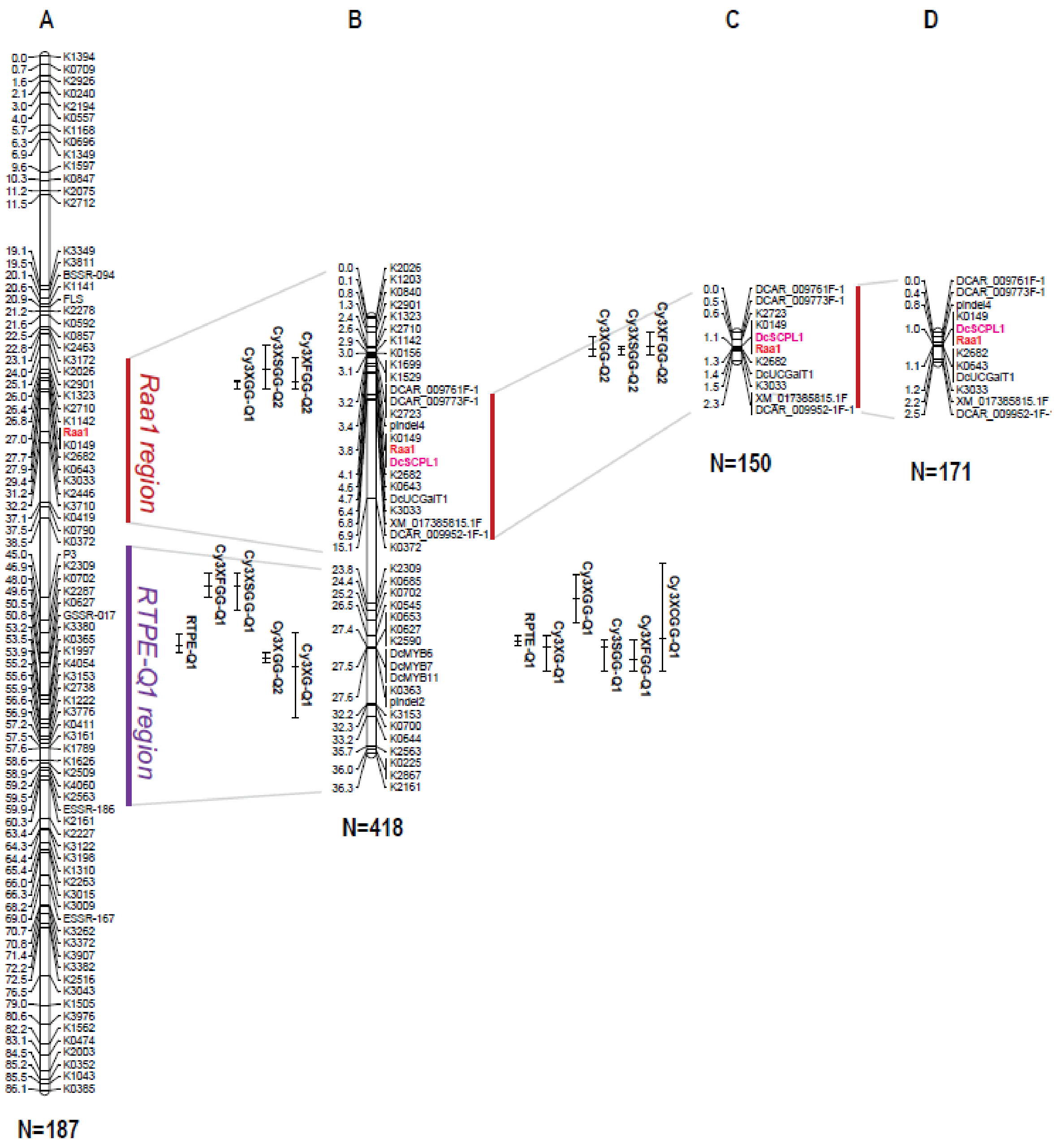

Supplementary Figure S3. Genetic mapping of anthocyanin pigment traits in chromosome 3 of different carrot populations. (**A**) QTLs for 'root total pigment estimate' (RTPE), root anthocyanin pigments (Cy3XG, Cy3XGG, Cy3XSGG, Cy3XFGG and Cy3XCGG) and acylation of root anthocyanins (*Raa1*), mapped in the original framework linkage map published by Cavagnaro et al. (2014). (**B**) High-resolution mapping in the RTPE-Q1 and Raa1 regions using a larger 70349 population. (**C-D**) Raa1 locus, conditioning anthocyanin acylation, highlighted in red and mapped in populations 5392 (**C**) and 5394 (**D**). Connecting dotted lines indicate the flanking markers of the map region further analyzed in this work. Bars to the right of the linkage groups represent support intervals of the QTL. Population size is indicated in parenthesis under each linkage group. The anthocyanin candidate acyltransferase *DcSPL1* and is denoted in bold purple letters.

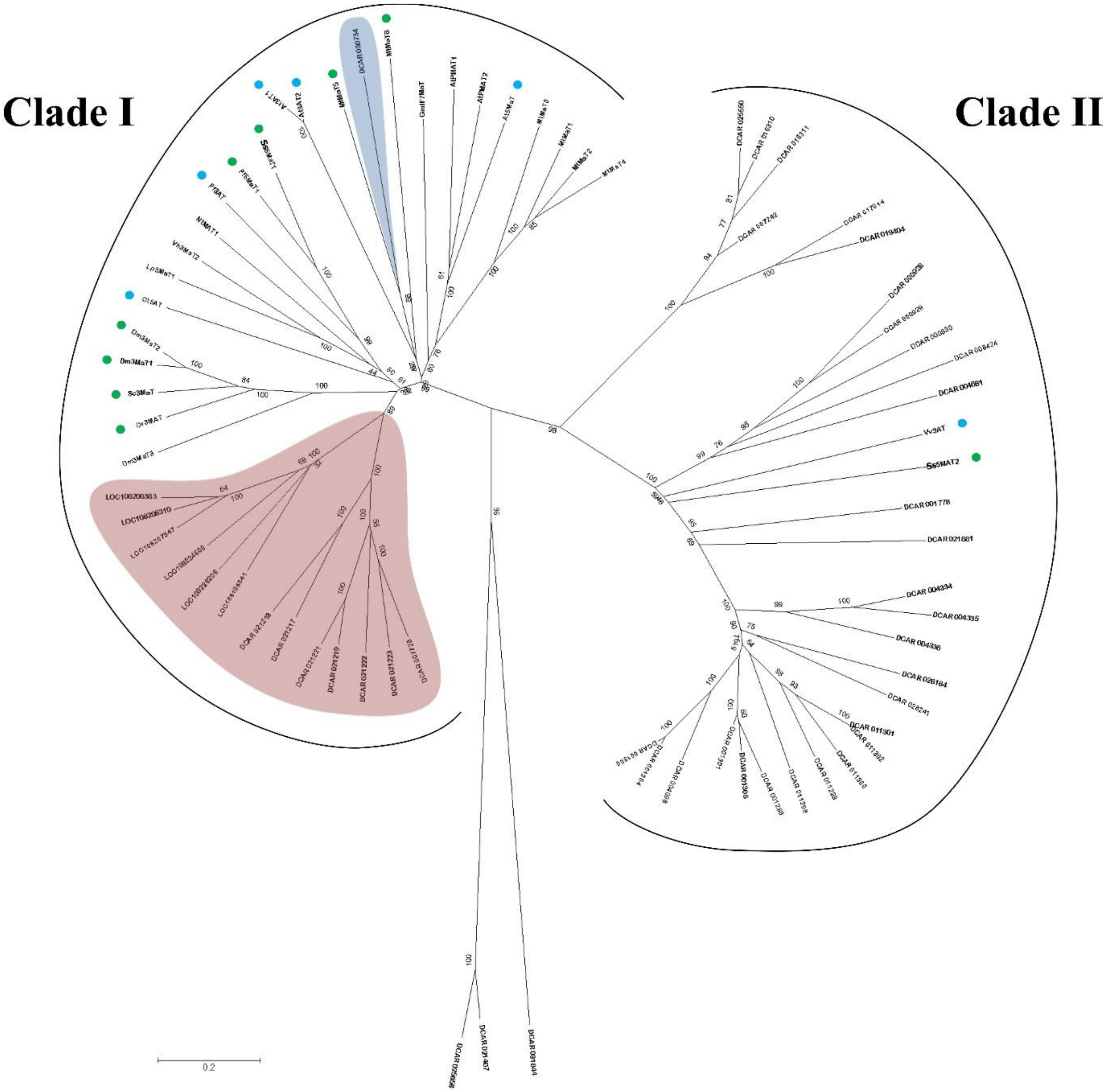

Supplementary Figure S4. Neighbor-joining phylogenetic analysis of BAHD proteins. Bootstrap values are percentage from 1000 replicates. The scale bar indicates 0.2 substitutions per site. Two clusters of carrot BAHD in clade I are highlighted in red and blue. Green circles indicate functionally characterized anthocyanin BAHD acyltransferase known to use manoyl-CoA as acyl donor, and blue circles indicate those using hydroxycinnamoyl-CoA as acyl donor. Protein sequences and complementary information are presented in **Supplementary Table S4** and **S5.** Functionally characterized flavonoid BAHD acyltransferases were collected from Bontpart et al. (2015): Flavonoid BAHD acyltranferases: Gt5AT, *Gentiana triflora* anthocyanin

5-aromatic acyltransferase; Dv3MAT, *Dahlia variabilis* malonyl-CoA: anthocyanidin 3-O-glucoside-6″-O-malonyltransferase; Sc3MAT, *Senecio cruentus* malonyl-CoA:anthocyanidin 3-O-glucoside 6″-O-malonyltransferase; Dm3MAT1-3, *Dendranthema morifolium* anthocyanidin 3-O-glucoside-6″-O-malonyltransferase 1-3; NtMAT1, *Nicotiana tabacum* malonyl-CoA flavonoid/naphthol glucoside acyltransferase 1; Vh3MAT1, *Verbena* hybridamalonyl-CoA:flavonol3-O-glucoside-6″-O-malonyltransferase; Lp3MAT1, *Lamium purpureum* malonyl-CoA:flavonol3-Oglucoside-6″-O-malonyltransferase 1; Pf3AT, *Perilla frutescens* hydroxycinnamoyl-CoA:anthocyanin 3-O-glucoside-6″-O-acyltransferase; Ss5MAT1, *Salvia splendens* malonyl-CoA:anthocyanin 5-O-glucoside-6″-O-malonyltransferase 1; Ss5MAT2, *Salvia splendens* anthocyanin 5-O-glucoside-4″-O-malonyltransferase 2; Pf5MAT, *Perilla frutescens* malonyl-CoA:anthocyanin 5-O-glucoside-6″-O-malonyltransferase; At5MAT, *Arabidopsis thaliana* malonyl-CoA:anthocyanidin 5-O-glucoside-6″-O-malonyltransferase; At3AT1-2, *Arabidopsis thaliana* hydroxycinnamoyl-CoA:anthocyanin 3-O-glucoside-6″-O-acyltransferase 1-2; GmIF7MAT, *Glycine max* malonyl-CoA:isoflavone 7-O-glucoside-6″-O-malonyltransferase; GmMT7, *Glycine max* malonyltransferase 7; MtMAT1-6, *Medicago truncatula* malonyl-CoA:flavonol 3-O-glucoside-6″-O-malonyltransferase 1-6; AtPMAT1-2, *Arabidopsis thaliana* phenolic glucoside malonyltransferase 1-2.

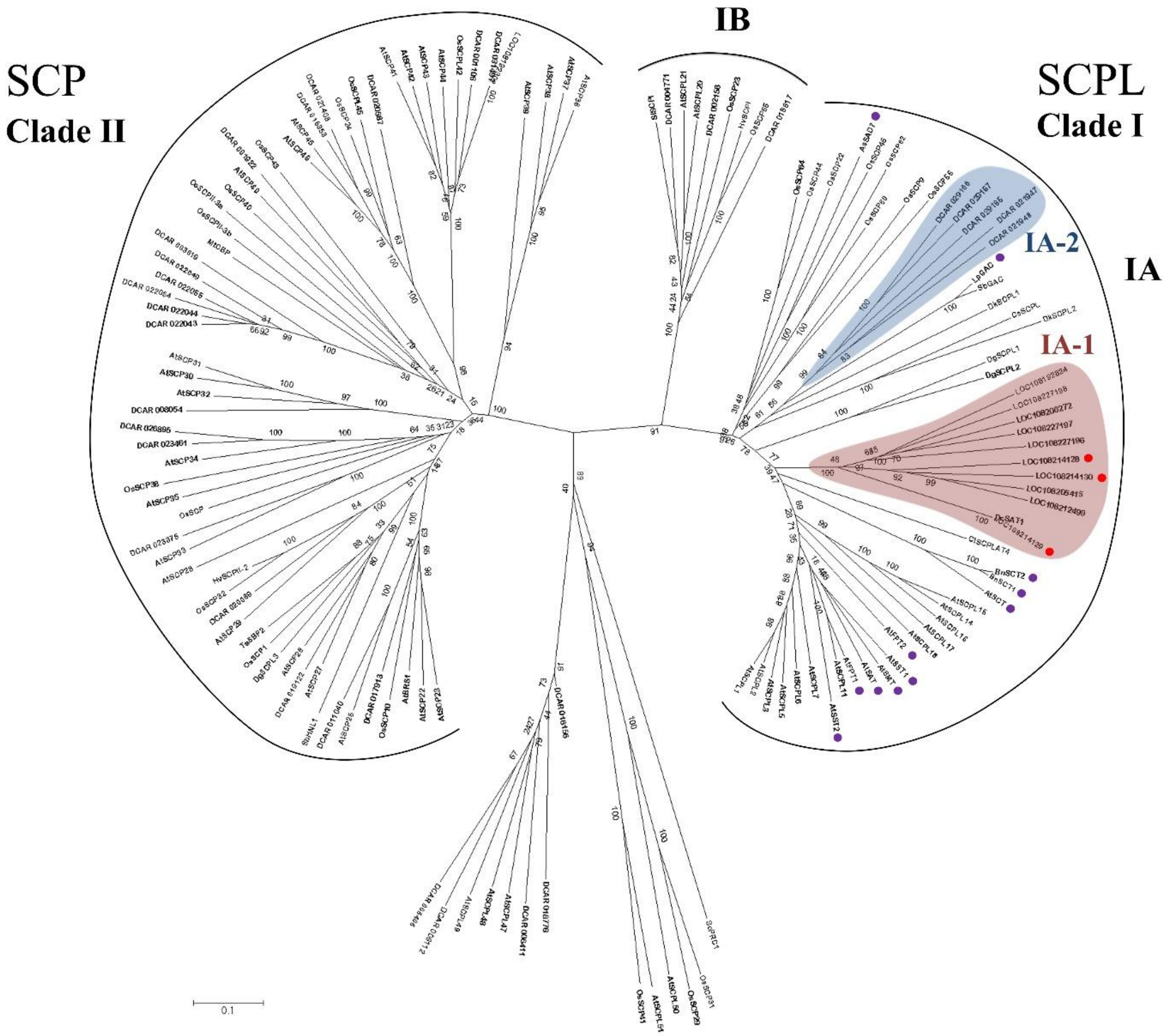

Supplementary Figure S5. Neighbor-joining phylogenetic analysis of SCP and SCPL proteins. Bootstrap values are percentage from 1000 replicates. The scale bar indicates 0.1 substitutions per site. Clades are labeled according to Fraser et al. (2005). Two clusters of carrot SCPL-ATs are highlighted, IA-1 (red) and IA-2 (blue). Protein sequences and complementary information are presented in **Supplementary Table S4** and **S5**. Red circles indicate *SCPL* genes located within the *Raa1* locus. Purple circles indicate functionally characterized SCPL-ATs.

A

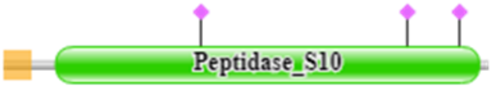

Peptidase_S10

B

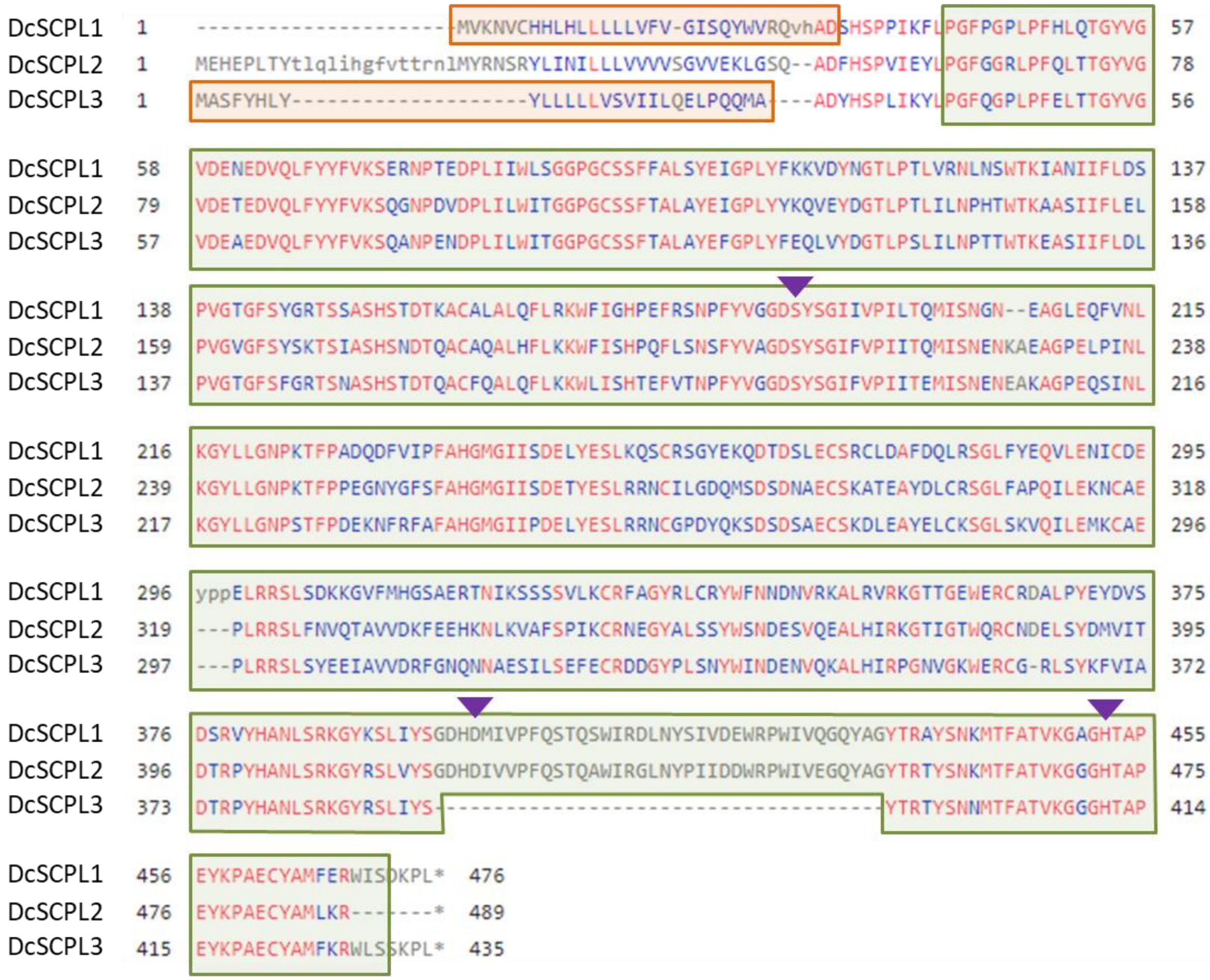

DcSCPL1 1 ----------------------MVKNVCHHLHLLLLLVFV-GISQYWVRQvhADSHSPPIKFLPGFPGPLPFHLQTGYVG 57
DcSCPL2 1 MEHEPLTYtlqlihgfvttrnlMYRNSRYLINILLLVVVVSGVVEKLGSQ--ADFHSPVIEYLPGFGGRLPFQLTTGYVG 78
DcSCPL3 1 MASFYHLY---------------------YLLLLLVSVIILQELPQQMA---ADYHSPLIKYLPGFQGPLPFELTTGYVG 56
DcSCPL1 58 VDENEDVQLFYYFVKSERNPTEDPLIIWLSGGPGCSSFFALSYEIGPLYFKKVDYNGTLPTLVRNLNSWTKIANIIFLDS 137
DcSCPL2 79 VDETEDVQLFYYFVKSQGNPDVDPLILWITGGPGCSSFTALAYEIGPLYYKQVEYDGTLPTLILNPHTWTKAASIIFLEL 158
DcSCPL3 57 VDEAEDVQLFYYFVKSQANPENDPLILWITGGPGCSSFTALAYEFGPLYFEQLVYDGTLPSLILNPTTWTKEASIIFLDL 136
DcSCPL1 138 PVGTGFSYGRTSSASHSTDTKACALALQFLRKWFIGHPEFRSNPFYVGGDSYSGIIVPILTQMISNGN--EAGLEQFVNL 215
DcSCPL2 159 PVGVGFSYSKTSIASHSNDTQACAQALHFLKKWFISHPQFLSNSFYVAGDSYSGIFVPIITQMISNENKAEAGPELPINL 238
DcSCPL3 137 PVGTGFSFGRTSNASHSTDTQACFQALQFLKKWLISHTEFVTNPFYVGGDSYSGIFVPIITEMISNENEAKAGPEQSINL 216
DcSCPL1 216 KGYLLGNPKTFPADQDFVIPFAHGMGIISDELYESLKQSCRSGYEKQDTDSLECSRCLDAFDQLRSGLFYEQVLENICDE 295
DcSCPL2 239 KGYLLGNPKTFPPEGNYGFSFAHGMGIISDETYESLRRNCILGDQMSDSDNAECSKATEAYDLCRSGLFAPQILEKNCAE 318
DcSCPL3 217 KGYLLGNPSTFPDEKNFRFAFAHGMGIIPDELYESLRRNCGPDYQKSDSDSAECSKDLEAYELCKSGLSKVQILEMKCAE 296
DcSCPL1 296 yppELRRSLSDKKGVFMHGSAERTNIKSSSSVLKCRFAGYRLCRYWFNNDNVRKALRVRKGTTGEWERCRDALPYEYDVS 375
DcSCPL2 319 ---PLRRSLFNVQTAVVDKFEEHKNLKVAFSPIKCRNEGYALSSYWSNDESVQEALHIRKGTIGTWQRCNDELSYDMVIT 395
DcSCPL3 297 ---PLRRSLSYEEIAVVDRFGNQNNAESILSEFECRDDGYPLSNYWINDENVQKALHIRPGNVGKWERCG-RLSYKFVIA 372
DcSCPL1 376 DSRVYHANLSRKGYKSLIYSGDHDMIVPFQSTQSWIRDLNYSIVDEWRPWIVQGQYAGYTRAYSNKMTFATVKGAGHTAP 455
DcSCPL2 396 DTRPYHANLSRKGYRSLVYSGDHDIVVPFQSTQAWIRGLNYPIIDDWRPWIVEGQYAGYTRTYSNKMTFATVKGGGHTAP 475
DcSCPL3 373 DTRPYHANLSRKGYRSLIYS-----------------------------------YTRTYSNNMTFATVKGGGHTAP 414
DcSCPL1 456 EYKPAECYAMFERWISDKPL* 476
DcSCPL2 476 EYKPAECYAMLKR-------* 489
DcSCPL3 415 EYKPAECYAMFKRWLSSKPL* 435

C

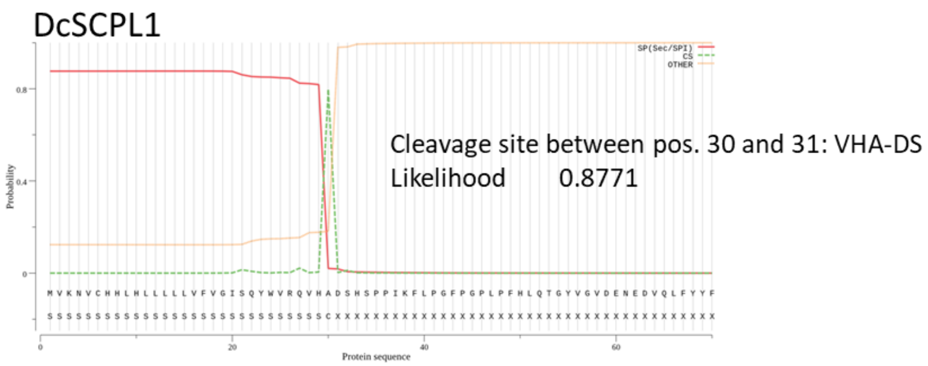

DcSCPL1
SP(Sec/SPI)
CS
OTHER
Cleavage site between pos. 30 and 31: VHA-DS
Likelihood 0.8771
Probability
0.8
0.4
0
Protein sequence
0
20
40
60

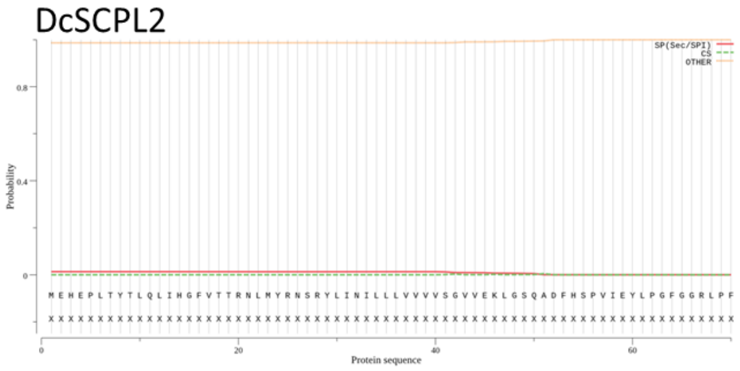

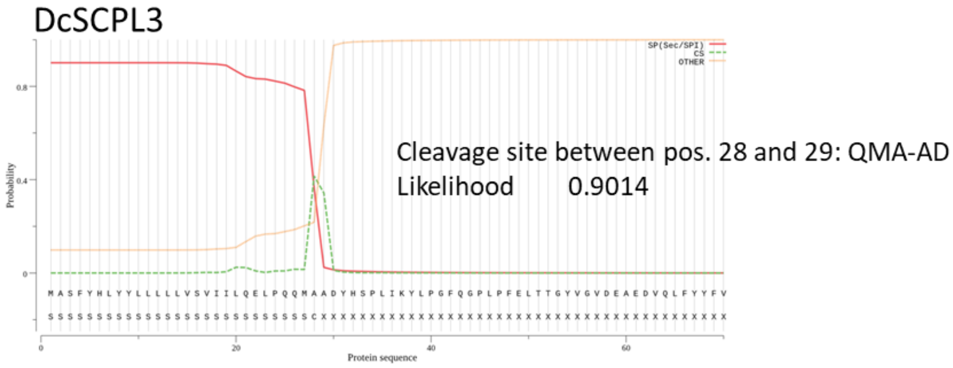

Supplementary Figure S6. DcSCPL1, DcSCPL2 and DcSCPL3 protein sequence analysis. (**A**) Predicted functional domains and signatures in DcSCPL1 amino acids sequence extracted from DH1 reference genome (Iorizzo et al., 2016) (https://pfam.xfam.org): the signal peptide (orange box), the Peptidase_S10 domain (green box) and the 3 amino-acids from the catalytic triad (purple arrows). (**B**) Protein alignment of DcSCPL1, DcSCPL2 and DcSCPL3, using COBALT (https://www.ncbi.nlm.nih.gov/tools/cobalt). Alignment columns are colored in blue when there is no gap and in red when all three residues are identical. The peptide signal is highlighted in orange, Peptidase_S10 domain is in green and the catalytic triad are marked with purple arrows. (**C**) Prediction of the N-terminal secretory signal peptide in DcSCPL1, DcSCPL2 and DcSCPL3, using SignalP-5.0 (http://www.cbs.dtu.dk/services/SignalP).

DcSCPL1-1

```
ATGGTGAAAAATGTATGCCACCATTTACATTTGTTGCTGCTGCTTGTTTTTGTTGTTGGGATTTCACAGTATTGGGTGAGGCAGGTGCATGCAGACTCTCAC
TCACCACCCATCAAGTTCCTTCCTGGTTTCCCAGGACCCCTCCCTTTTCATCTGCAAACTGG___GTATGTGGGGGTGGATGAAAATGAAGATGTGCAATTG
TTCTATTATTTTGTCAAGTCGGAGAGAAATCCAACAGAGGACCCTCTTATTATTTGGCTTTCAGGAGGCCCTGGCTGTTCTTCCTTCTTTGCCCTTTCTTAT
GAAATTG___GTCCGTTATATTTTAAGAAAGTAGACTACAACGGAACCTTGCCAACCCTAGTTCGAAATCTCAATTCATGGACTAAG___ATAGCAAATATA
ATATTTCTGGATTCTCCGGTCGGTACTGGTTTTTCATATGGCAGGACATCCTCTGCTTCTCACTCTACTGACACCAAAGCATGTGCTCTAGCCCTCCAGTTC
TTGAGGAAG___TGGTTCATTGGTCATCCTGAATTCCGTTCAAATCCATTCTATGTTGGTGGAGACTCTTACTCTGGAATTATTGTTCCGATTCTCACTCAA
ATGATCTCAAACG___GAAACGAAGCAGGCCTTGAACAATTTGTCAATCTCAAG___GGTTACTTGCTTGGAAATCCTAAAACATTCCCCGCTGACCAAGAC
TTTGTTATCCCTTTTGCTCATGGTATGGGTATTATTTCGGATGAACTATATGAG___TCATTGAAGCAGAGTTGTAGATCTGGCTATGAGAAGCAAGATACT
GACAGTTTAGAGTGTTCACGTTGTTTAGATGCTTTTGATCAG___TTGCGCAGTGGACTTTTCTATGAACAAGTTCTGGAGAATATATGTGATGAATATCCA
CCCGAGCTGCGAAGGTCACTGTCTGATAAAAAAGAAGTTTTTATGCATGGATCTGCAGAGAGAAATAATATTAAGTCATCCTCTTCTGTACTTAAATGTCGT
___TTTGCTGGGTACCGGCTTTGTAGATACTGGTTTAACAACGATAATGTCCGGAAAGCACTGCATGTCAGAAAG___GGGACTACAGGGGAATGGGAAAGA
TGCAGAGATGCTTTGCCTTACGAATATGATGTTTCGGATAGTAGAGTGTATCATGCAAATCTAAGCAGGAAGGGCTATAAATCTCTAATATACAG___TGGT
GACCATGACATGATCGTTCCATTTCAATCAACTCAGTCTTGGATTAGAGATCTGAACTACTCCATTGTAGATGAATGGCGACCATGGATTGTCCAAGGCCAA
TATGCTGG___TTACACTAGGGCTTACTCCAACAAAATGACGTTCGCTACTGTTAAG___GGCGCAGGCCATACAGCTCCGGAATACAAGCCTGCAGAATGT
TATGCTATGTTTGAAAGATGGATATCCGACAAACCTCTGTAA
```

```
M V K N V C H H L H L L L L L V F V V G I
S Q Y W V R Q V H A D S H S P P I K F L
P G F P G P L P F H L Q T G Y V G V D E
N E D V Q L F Y Y F V K S E R N P T E D
P L I I W L S G G P G C S S F F A L S Y E
I G P L Y F K K V D Y N G T L P T L V R N
L N S W T K I A N I I F L D S P V G T G F
S Y G R T S S A S H S T D T K A C A L A
L Q F L R K W F I G H P E F R S N P F Y
V G G D S Y S G I I V P I L T Q M I S N G
N E A G L E Q F V N L K G Y L L G N P K
T F P A D Q D F V I P F A H G M G I I S D
E L Y E S L K Q S C R S G Y E K Q D T D
S L E C S R C L D A F D Q L R S G L F Y
E Q V L E N I C D E Y P P E L R R S L S
D K K E V F M H G S A E R N N I K S S S
S V L K C R F A G Y R L C R Y W F N N D
N V R K A L H V R K G T T G E W E R C
R D A L P Y E Y D V S D S R V Y H A N L
S R K G Y K S L I Y S G D H D M I V P F
Q S T Q S W I R D L N Y S I V D E W R P
W I V Q G Q Y A G Y T R A Y S N K M T F
A T V K G A G H T A P E Y K P A E C Y
A M F E R W I S D K P L Stop
```

DcSCPL1-2

```
ATGGTGATGAACGTATGCCACCATTTACATTTGTTGCTGCTGCTTGTTTTTGTTGTTGGGATTTCACAGTATTGGGTGAGGCAGGTGCATGCAGACTCTCAC
TCACCGCCCATCAAGTTCCTTCCTGGTTTCCCAGGACCCCTTCCTTTTCATCTGCAAACTGG___GTATGTGGGGGTGGATGAAAATGAAGATGTGCAATTG
TTCTATTATTTTGTCAAGTCGGAGAGAAATCCAACAGAGGACCCTCTTATTATTTGGCTTTCAGGAGGCCCTGGCTGTTCTTCCTTCTTTGCCCTTTCTTAT
GAAATTG___GTCCGTTATATTTTAAGAAAGTAGACTACAACGGAACCTTGCCAACCCTAGTTCGAAATCTCAATTCATGGACTAAG___ATAGCAAATATA
ATATTTCTGGATTCTCCGGTCGGTACTGGTTTTTCATATGGCAGGACATCCTCTGCTTCTCACTCTACTGACACCAAAGCATGTGCTCTAGCCCTCCAGTTC
TTGAGGAAG___TGGTTCATTGGTCATCCTGAATTCCGTTCAAATCCATTCTATGTTGGTGGAGACTCTTACTCTGGAATTATTGTTCCGATTCTCACTCAA
ATGATCTCAAACG___GAAACGAAGCAGGCCTTGAACAATTTGTCAATCTCAAG___GGTTACTTGCTTGGAAATCCTAAAACATTCCCCGCTGACCAAGAC
TTTGTTATCCCTTTTGCTCATGGTATGGGTATTATTTCGGATGAACTATATGAG___TCATTGAAGCAGAGTTGTAGATCTGGCTATGAGAAGCAAGATACT
GACAGTTTAGAGTGTTCACGTTGTTTAGATGCTTTTGATCAG___TTGCGCAGTGGACTTTTCTATGAACAAGTTCTGGAGAATATATGTGATGAATATCCA
CCCGAGCTGCGAAGGTCACTGTCTGATAAAAAAGAAGTTTTTATGCATGGATCTGCAGAGAGAACTAATATTAAGTCATCCTCTTCTGTACTTAAATGTCGT
___TTTGCTGGGTACCGGCTTTGTAGATACTGGTTTAACAACGATAATGTCCGGAAAGCACTGCATGTCAGAAAG___GGGACTACAGGGGAATGGGAAAGA
TGCAGAGATGCTTTGCCTTATGAATATGATGTTTCGGATAGTAGAGTGTATCATGCAAATCTAAGCAGGAAGGGCTATAAATCTCTAATATACAG___TGGT
GACCATGACATGATCGTTCCATTTCAATCAACTCAGTCTTGGATTAGAGATCTGAACTACTCCATTGTAGATGAATGGCGACCATGGATTGTCCAAGGCCAA
TATGCTGG___TTACACTAGGGCTTACTCCAACAAAATGACGTTCGCTACTGTTAAG___GGCGCAGGCCATACAGCTCCGGAATACAAGCCTGCGGAATGT
TATGCTATGTTTGAAAGATGGATATCCGACAAACCTCTGTAA
```

```
M V M N V C H H L H L L L L L V F V V G I
S Q Y W V R Q V H A D S H S P P I K F L
P G F P G P L P F H L Q T G Y V G V D E
N E D V Q L F Y Y F V K S E R N P T E D
P L I I W L S G G P G C S S F F A L S Y E
I D S K Y N I S G F S G R Y W F F I W Q
D I L C F S L Y Stop
```

Supplementary Figure S7. CDS and protein sequences of *DcSCPL1* alleles present in 5394 plants. CDS sequence (left panel) and its corresponding amino acid sequence (right panel) are indicated for both *DcSCPL1* alleles: *DcSCPL1-1* (dominant allele) and *DcSCPL1-2* (recessive allele). Polymorphisms between alleles are highlighted in blue for the substitutions and in red for the deletion. Junctions between exons are marked with underscores. Amino acids composing the catalytic triad (Ser-Asp-His) are highlighted in green.

ATTCATTACCTGACCTCGGTTATTTTGGCAGTCACATTGATAATGTATAAAGTTTAACTGCAGGTCCGTTATATTTTAAGAAAGTAGACTACAACGGAACCTTG
CCAACCCTAGTTCGAAATCTCAATTCATGGACTAAG**GTAGGGCTGCACATGGGCTGGGGTTGGGTTGGGTTTAACCATTAAGGTAACCCAACCCATTTAGTTCG**
GGTCACAAAAAAATAACCCATTAATATCAAAAAAATCCTATAACCCAATTTTTACTACTTATGTGATGGGTTGGGTCGGGTTGGTTTGGGTTGAATGGGTTGAC
AAAGCAAAAAAGTTTCCATAACAATTATATCTTCACAAGCTAAGTAGCTAACATTGACTCTAGGTGAATAAAGTCCAACAAGTTAAAGAAAAGAGAAAGTCCAT
ACTGCAAGTTAAAAGAAAGAAGTGTTGCTAATGACCGGCATTAGTAGTTTGTTAATCAAATGTCTCAATATGAAAATTTATCTGATGTATAATACCGGAAGAGT
GACATATTATTTATATGGACTGTTTATCACAGTTAAtAACGaACCTTATtAACTTCCTACTAGATGGAGTTTACAACCTAATAGATGCAAATTTAATACTACTA
CTATATCTTATTTACTCTTTATAATAGTTGGTTGGGTGGTTGGTTAGGTTTAAAAGAGTAAAATTTGCCCACACCCCAAGTGTACCGGTCTCATTGAAAATTAC
CCAAACTACCCATGGGTTGGAGTGGTTCGGTTTGAACCGCGCCTATATTAAGGGTTGGGTGTGGTTCGGTTTTGGCGGGTTCGGTTGCCCCTGGAGCCAGCCCT
AGACTAAGGTGTGTTATCTGCTTGAAATACCAAGTAATATTTTACGTATGCAATACAAGAACCCACCATCCTCAACGGAAAAGCATGTTCCCATCACGATTGTC
TTAGCTCTTGTAACAGATTTTGCATATATTATGTGATCAGAATTTTCCAAATTTTGAGTTGTAGTTGCAAATGAAACACTTCTGATTAGTATGATTTCTTACTG
TTTAGCTCTTGGTAGCTAGTGAATTAATACATAACCTTTTAACGAAAAACCTGACTTTATTATGCTCATTTGCCTCCATTTTAACTTAGAAAGTTCTCAAATGT
TGATTTGCATAAATAATTTCAACGACTTCAATCTCATTACTACTCCACTGTTTATAAATGTTAAAAGGTTGGAATCAAATTTCACGTTTTGTAATGTTTGTTTA
TAAGGATTATAATTTAGCTATTGATAGTATTATTCCTTTTATCTCATACGACAGACACAGATGAACTCCCAGTTGGGTATTGATATATGAATGATGATTTTGAT
ATATGGTGAGAATCAATTTACAGATAGCAAATATAATATTTCTGGATTCTCCGGTCGGTACTGGTTTTTCATATGGCAGGACATCCTCTGCTTCTCACTCTACT
GACACCAAAGCATGTGCTCTAGCCCTCCAGTTCTTGAGGAAGGTACGGATCTAATGATCATCCGTGTATAATGTATCATTCAGCTATTCATACATTTGTTGTCT
GCATTCAAGGTATTAATTTAATGTCTAACAAAATTATGATTTT

Supplementary Figure S8. Genomic sequence of *DcSCPL1-2* in 5394-PR-LAA plants. Sequence from the 3rd to the 4th exons of *DcSCPL1*. Exons are underlined. The green sequence corresponds to a 700 bp fragment, absent in *DcSCPL1-1* allele, which is inserted at the exon-3/intron-3 junction.

A

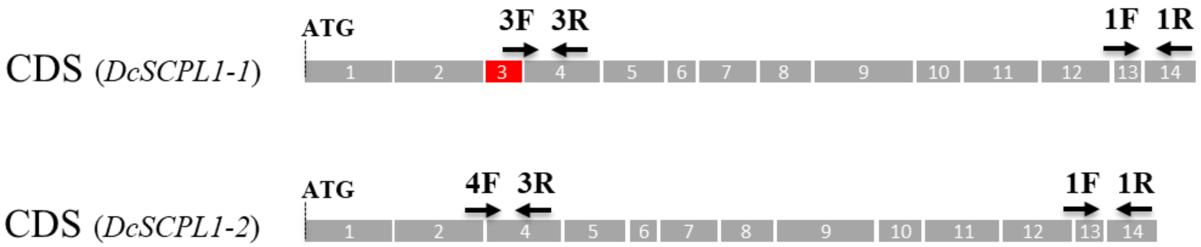

B

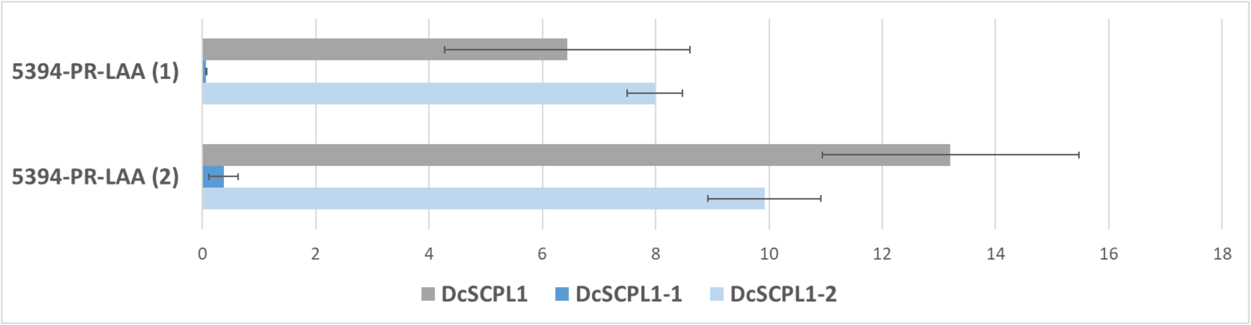

Supplementary Figure S9. Relative expression of *DcSCPL1* alleles in 5394-PR-LAA plants. (**A**) Position of the primers used for the detection of *DcSCPL1* (1F/1R), *DcSCPL1-1* (3F/3R) and *DcSCLP1-2* (4F/3R). (**B**) Relative expression level of *DcSCPL1*, *DcSCPL1-1* and *DcSCPL1-2*, in two 5394-PR-LAA homozygous plants, detected by RT-qPCR.

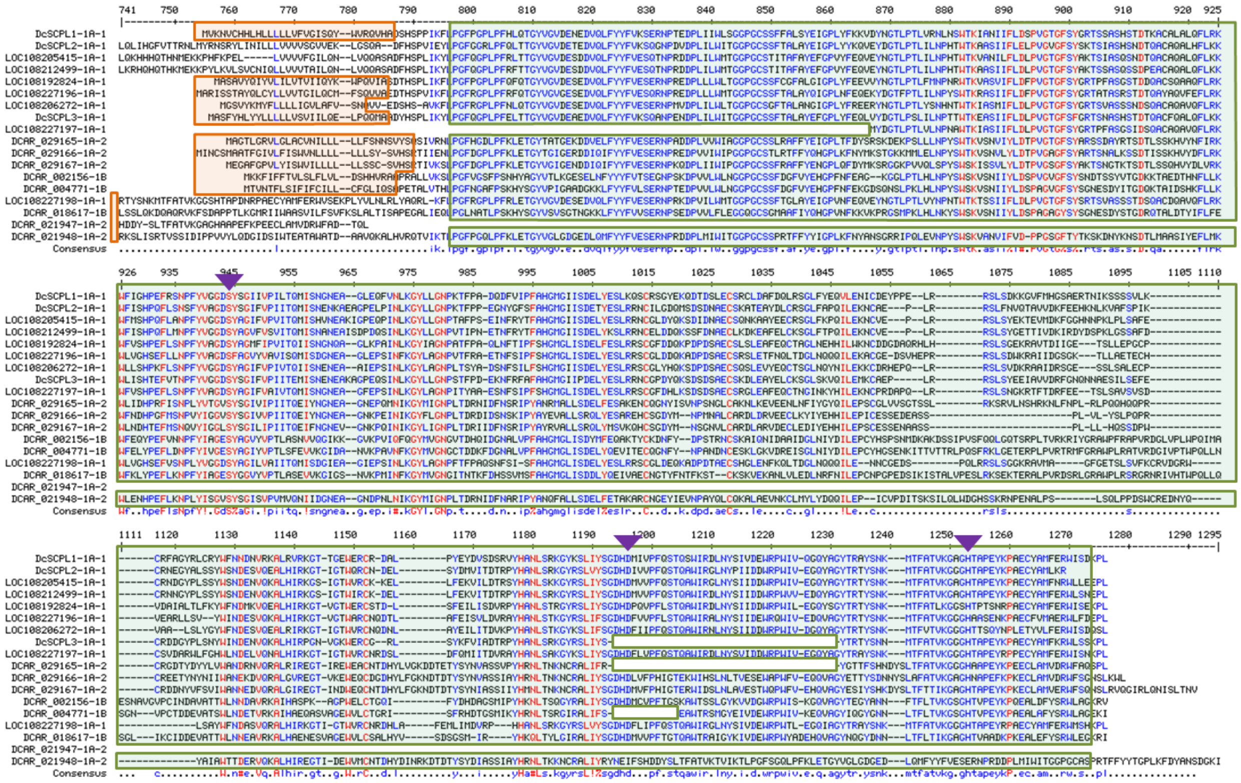

Supplementary Figure S10. Alignment of putative carrot SCPL acyltransferases. Alignment between DcSCPL sequences from clusters IA and IB (**Supplementary Table S5**), made using MultAlin (http://multalin.toulouse.inra.fr). Alignment columns are colored in red when residues are above 90% identical and in blue when they are 50-90% identical. The approximate position of the signal peptide is framed in orange and the Peptidase S10 domain is framed in green. The position of amino acids from the catalytic triad are indicated with a purple arrows.

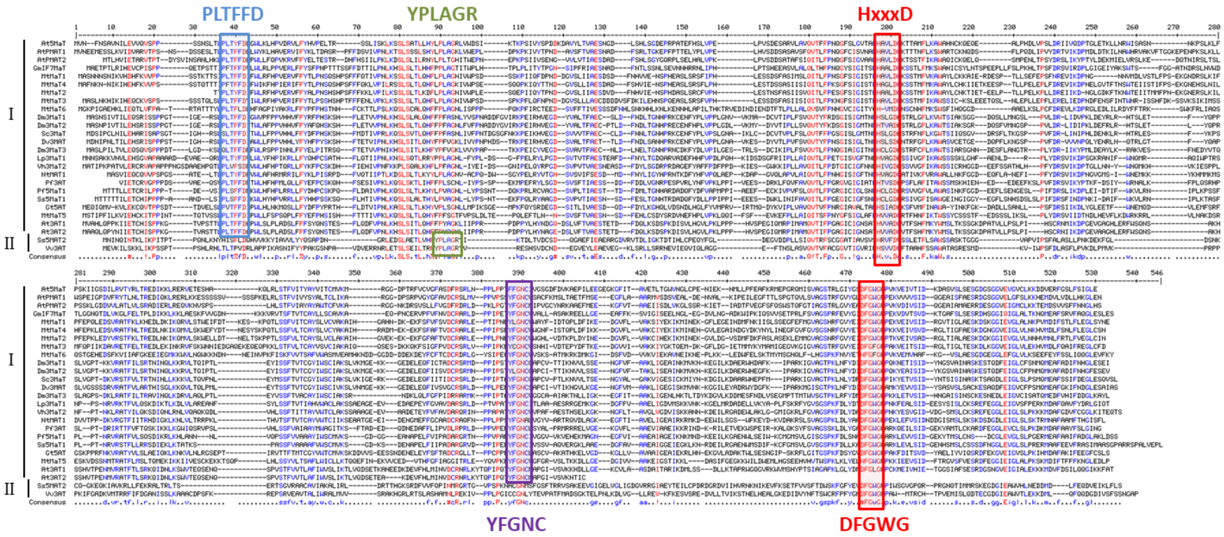

Supplementary Figure S11. Alignment of previously characterized flavonoid BAHDs. Alignment between characterized flavonoid BAHD sequences present in cluster I and II (**Supplementary Table S4**), made using MultAlin (http://multalin.toulouse.inra.fr). Alignment columns are colored in red when residues are above 90% identical and in blue when they are 50-90% identical. Conserved motifs are highlighted as follow: BAHD specific (red), clade I specific (blue), anthocyanin specific (purple) and conserved outside clade I (green).

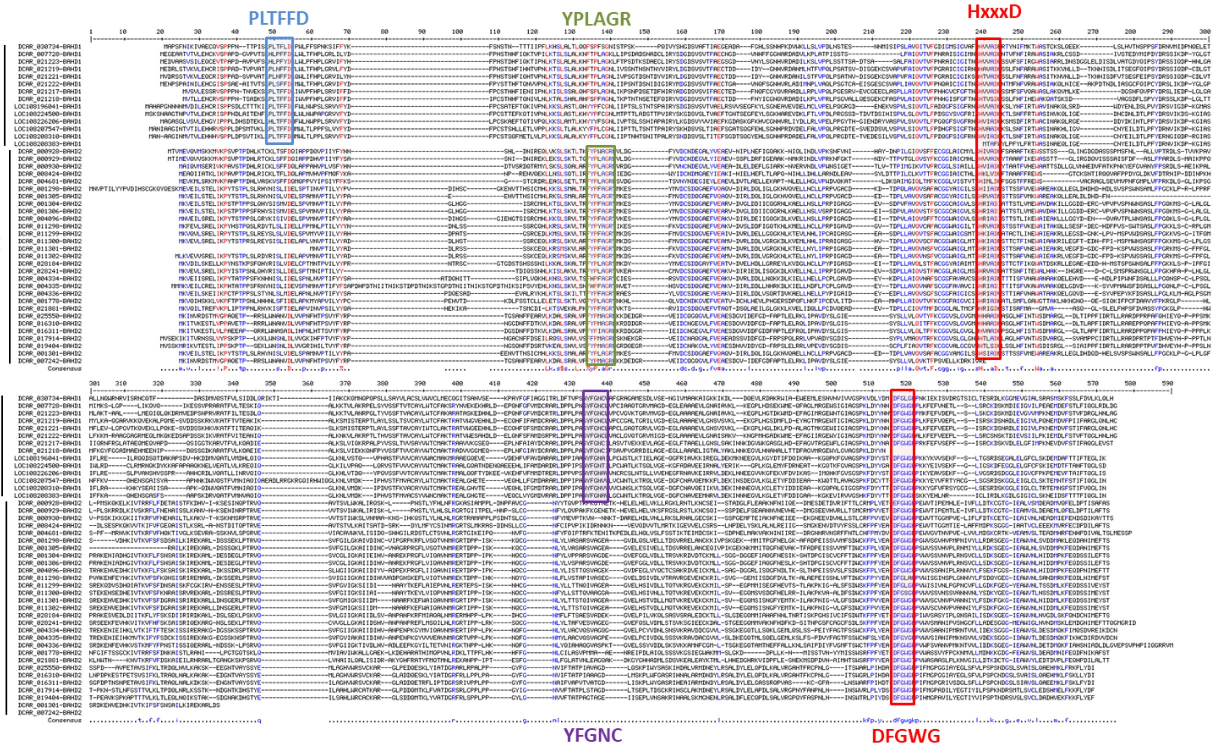

Supplementary Figure S12. Alignment of carrot BAHDs. Alignment between DcBAHD sequences from cluster I and II (**Supplementary Table S5**), made using MultAlin (http://multalin.toulouse.inra.fr). Alignment columns are colored in red when residues are above 90% identical and in blue when they are 50-90% identical. Conserved motifs are highlighted as follow: BAHD specific (red), clade I specific (blue), anthocyanin specific (purple) and conserved outside clade I (green).
